# From a Multi-Omics Signature to a Therapeutic Candidate: Computational Prediction and Experimental Validation in Liver Fibrosis

**DOI:** 10.3390/ph19030495

**Published:** 2026-03-17

**Authors:** Yingying Qin, Shuoshuo Ma, Haoyuan Hong, Deyuan Zhong, Yuxin Liang, Yuhao Su, Yahui Chen, Xing Chen, Yizhun Zhu, Xiaolun Huang

**Affiliations:** 1 School of Pharmacy, Faculty of Medicine, Macau University of Science and Technology, Avenida WaiLong, Taipa, Macau SAR 999078, China; 2 Liver Transplantation Center and HBP Surgery, Sichuan Clinical Research Center for Cancer, Sichuan Cancer Hospital & Institute, Sichuan Cancer Center, School of Medicine, University of Electronic Science and Technology of China, Chengdu 610041, China; 3 School of Pharmacy, Faculty of Medicine & State Key Laboratory of Quality Research in Chinese Medicines, Macau University of Science and Technology, Avenida WaiLong, Taipa, Macau SAR 999078, China

**Keywords:** liver fibrosis, bioinformatic analysis, machine learning, cross-etiology, Withaferin A, drug repositioning

## Abstract

**Background**: Advanced liver fibrosis (LF) is a major determinant of prognosis across chronic liver diseases. Current biomarkers are often etiology-specific and lack cross-cohort robustness. Shared molecular drivers across etiologies remain incompletely defined, and effective anti-fibrotic therapies are limited. **Methods**: We developed a multi-algorithm consensus machine-learning framework to derive a robust LF progression signature. In the training non-alcoholic fatty liver disease (NAFLD) cohort GSE213621 (*n* = 368), samples were formulated as a binary classification task (mild fibrosis, F0–F2; advanced fibrosis, F3–F4). Candidate genes were screened in parallel using Boruta, Least Absolute Shrinkage and Selection Operator (LASSO), random forest, and eXtreme Gradient Boosting (XGBoost). Genes selected by at least two algorithms were defined as a high-consensus pool, and genes consistently selected by all four algorithms were prioritized to construct a core signature. Model performance was evaluated by stratified cross-validation in the training cohort and externally validated in four independent cohorts of different etiologies (GSE49541, GSE84044, GSE130970, and GSE276114). Cellular sources of signature genes were characterized using single-cell RNA sequencing (scRNA-seq) datasets GSE136103 (human) and GSE172492 (mouse). For therapeutic discovery, the high-consensus expression profile was queried against the Connectivity Map (CMap) to prioritize compounds predicted to reverse the fibrotic transcriptional program. Withaferin A (WFA) was selected for experimental validation in a carbon tetrachloride (CCl_4_)-induced mouse LF model and in the transforming growth factor-β1 (TGF-β1)-stimulated human hepatic stellate cell line LX-2. Bulk liver RNA-seq profiling was performed to interrogate WFA-associated molecular changes in vivo. **Results**: We identified a six-gene signature (CLEC4M, COL25A1, ITGBL1, NALCN, PAPPA, and PEG3) that discriminated advanced from mild fibrosis, achieving a mean AUC of 0.890 in internal cross-validation and an average AUC of 0.864 across external validation cohorts. scRNA-seq analysis revealed cell-type-specific expression with prominent enrichment in fibroblast populations. In vivo, WFA markedly attenuated CCl_4_-induced fibrosis (*p* < 0.05) and reversed 1314 fibrosis-associated differentially expressed genes (adjusted *p* < 0.05), which were enriched in fatty acid metabolism and PPAR signaling, as well as extracellular matrix (ECM)–receptor interaction and focal adhesion (adjusted *p* < 0.05). In vitro, WFA suppressed TGF-β1-induced LX-2 activation, reducing α-SMA and Fibronectin expression (*p* < 0.05). **Conclusions**: We report a six-gene signature that robustly predicts advanced LF across etiologies, define its cellular context using single-cell atlases, and validate the anti-fibrotic activity of WFA in both in vivo and in vitro models. Bulk liver RNA-seq and cellular evidence further suggest that WFA-associated effects are linked to lipid metabolic programs, ECM remodeling, and attenuation of hepatic stellate cell activation.

## 1. Introduction

Liver fibrosis (LF) is a common pathological consequence of chronic liver diseases, including viral hepatitis (e.g., hepatitis B virus [HBV] infection) and non-alcoholic fatty liver disease (NAFLD), characterized by excessive extracellular matrix (ECM) deposition and progressive architectural distortion in response to persistent hepatic injury [1].

At the population level, chronic liver diseases remain a leading cause of premature mortality, accounting for approximately 2 million deaths annually worldwide (about 4% of all deaths) [2]. Consistent with these estimates, the Global Burden of Disease (GBD) 2021 study reported 1,425,142 deaths attributable to cirrhosis and other chronic liver diseases in 2021 [3,4]. In parallel, primary liver cancer continues to impose a substantial burden; based on GLOBOCAN 2022 estimates, liver cancer accounted for 866,136 new cases and 758,725 deaths worldwide in 2022 [5,6]. Metabolic liver disease is a key driver of this trend. NAFLD, recently renamed metabolic dysfunction-associated steatotic liver disease (MASLD) [7], affects approximately 30% of adults globally [8], and population-level evidence from mainland China similarly suggests a prevalence of around 30% with a rapid increase over the past decade [9,10].

Compared to mild LF (stages F0–F2), progression to advanced LF (stages F3–F4) [11] markedly increases the risk of decompensated cirrhosis and hepatocellular carcinoma (HCC) [12,13], posing a major global health burden [14]. Clinically, the transition from bridging fibrosis to cirrhosis represents a pivotal inflection point; longitudinal studies in NAFLD/non-alcoholic steatohepatitis (NASH) cohorts consistently demonstrate substantially higher cumulative risks of liver-related events once cirrhosis is established [15]. These observations support the use of F3–F4 as a clinically meaningful endpoint for risk stratification and therapeutic decision-making.

Although etiological control—such as antiviral therapy for specific causes—can effectively reduce disease activity, therapeutic agents that directly halt or reverse the fibrotic process itself remain lacking [16]. Despite substantial progress in mechanistic understanding, no broadly effective, etiology-agnostic anti-fibrotic therapy has been established in routine clinical practice, and most strategies remain investigational [17,18]. This unmet clinical need highlights the urgency of developing etiology-agnostic therapeutic strategies [19].

High-throughput transcriptomics has revolutionized the systematic exploration of LF. However, many previously reported molecular signatures are limited by reliance on single-etiology cohorts or single feature-selection methods, which often compromised robustness and generalizability across diverse populations. An ideal, clinically translatable molecular signature should be derived from pathogenic mechanisms shared across etiologies and should be rigorously validated through multi-algorithm consensus screening and independent multi-cohort verification to ensure reliability [20,21]. Mechanistically, fibrosis represents a convergent wound-healing program across etiologies, in which hepatic stellate cell (HSC) activation and ECM remodeling constitute central pathogenic nodes [22]. Therefore, consensus-driven feature selection that explicitly accounts for etiologic heterogeneity is essential for generating signatures that are both reproducible and clinically portable.

Moreover, a robust disease molecular signature can serve as a “fingerprint” of the core pathological network and directly drive therapeutic discovery. Computational drug repositioning approaches, such as the Connectivity Map (CMap) platform, can systematically predict compounds with the potential to “reverse” disease-associated transcriptional programs by matching disease signatures with drug-induced gene expression profiles [23], thereby bridging molecular diagnostics and treatment discovery. The CMap was originally introduced to connect drugs, genes, and disease states through shared gene-expression signatures [24] and was later expanded at scale via the L1000 platform to enable high-throughput, systematic perturbational mapping [25].

To address the critical need for robust, etiology-agnostic tools in advanced LF, this study aims to bridge the gap between molecular profiling and therapeutic discovery. We first developed an integrated multi-algorithm machine-learning pipeline to identify a highly robust gene molecular signature from NAFLD cohort data. Its predictive performance and biological relevance were validated across multiple independent cohorts and further contextualized at single-cell resolution, ensuring its robustness and clinical portability. Leveraging this signature, we then employed the CMap platform to prioritize candidate therapeutic compounds. Finally, we evaluated the anti-fibrotic and curative efficacy and explored the associated molecular changes of the candidate, Withaferin A (WFA), using both an in vivo carbon tetrachloride (CCl_4_)-induced mouse LF model and in vitro assays. We aim to deliver a diagnostic signature for LF and demonstrate a practical translational pathway from computational prediction to experimental verification.

## 2. Results

### 2.1. Identification of Molecular Features in LF Progression via Multi-Algorithm Consensus Machine Learning

**Figure 1:**
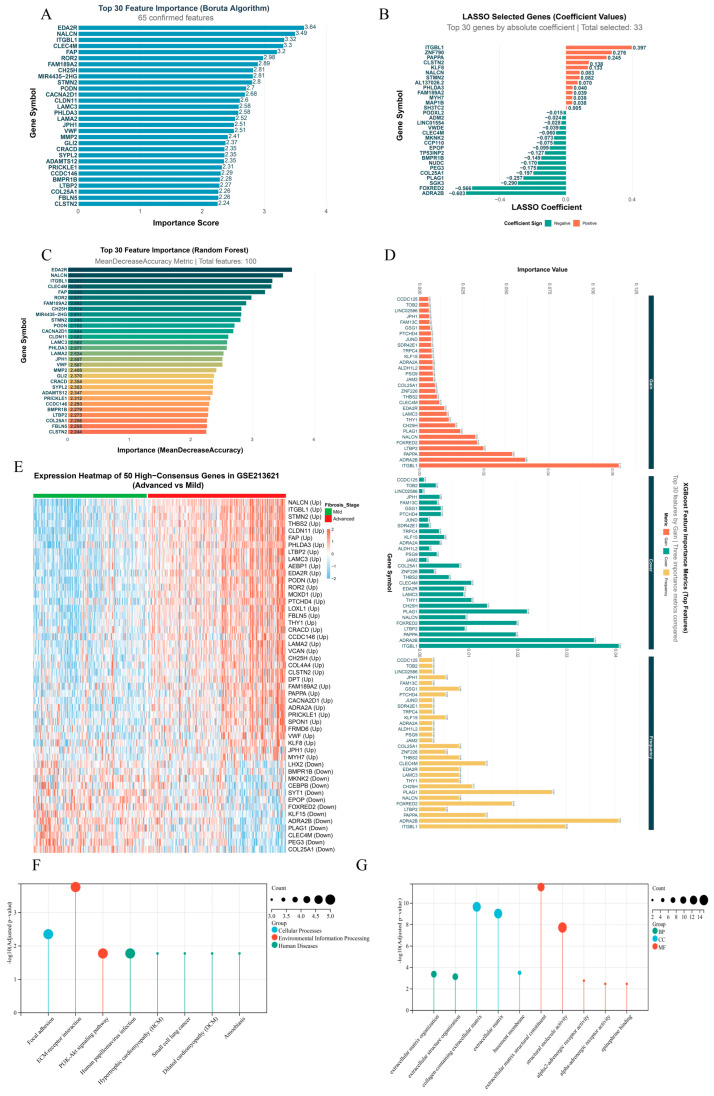
Identification of molecular features in LF progression using a multi-algorithm consensus machine-learning framework. (**A**) Top 30 Boruta-confirmed genes ranked by importance (Z-score). Boruta was run for 200 iterations and confirmed 65 genes exceeding shadow-feature importance. (**B**) Top 30 LASSO-selected genes ranked by absolute coefficient under the optimal penalty (lambda.1se; 10-fold cross-validation); 33 genes had non-zero coefficients. (**C**) Top 30 Random Forest features ranked by mean decrease in accuracy; the model (500 trees) identified 100 important genes. (**D**) Top 30 XGBoost features ranked by importance metrics (gain, cover, frequency); 50 genes with above-average gain were retained. (**E**) Heatmap showing standardized expression of 50 high-consensus genes (selected by ≥2 algorithms) in GSE213621, with samples grouped by fibrosis stage. (**F**) KEGG pathway enrichment of the 50 high-consensus genes. (**G**) GO cellular component enrichment of the 50 high-consensus genes.

By applying four complementary machine-learning algorithms in parallel to the GSE213621 training cohort, we identified gene features associated with advanced LF. Boruta confirmed 65 significant genes, with EDA2R, NALCN, and ITGBL1 ranking highest by Z-score (Figure 1A). Least Absolute Shrinkage and Selection Operator (LASSO) regression selected 33 genes with non-zero coefficients, among which NALCN, ITGBL1, and CLEC4M showed the largest absolute coefficients (Figure 1B). Random Forest identified 100 important genes (Figure 1C), and eXtreme Gradient Boosting (XGBoost) selected 50 genes based on integrated importance metrics, including gain, cover, and frequency (Figure 1D). The number of selected features and the key settings for each algorithm are summarized in Table 1.

To mitigate algorithm-specific bias, we defined high-consensus genes as those selected by at least two algorithms, yielding a final set of 50 genes. This consensus set displayed a stage-dependent expression pattern in the training cohort, clearly separating mild from advanced fibrosis samples (Figure 1E). Functional enrichment analyses supported the biological relevance of these genes. Kyoto Encyclopedia of Genes and Genomes (KEGG) analysis showed significant enrichment in canonical fibrosis-related pathways, including ECM–receptor interaction, focal adhesion, and PI3K–Akt signaling (Figure 1F). Gene Ontology (GO) cellular component analysis indicated predominant localization to the collagen-containing ECM and basement membrane, consistent with roles in ECM remodeling (Figure 1G). Notably, enrichment for terms related to adrenergic receptor activity suggests a potential contribution of neuroendocrine signaling to the fibrotic microenvironment.

### 2.2. The Six-Gene Signature Demonstrates Robust Predictive Power Across Etiologies and Consistent Expression Patterns

**Figure 2:**
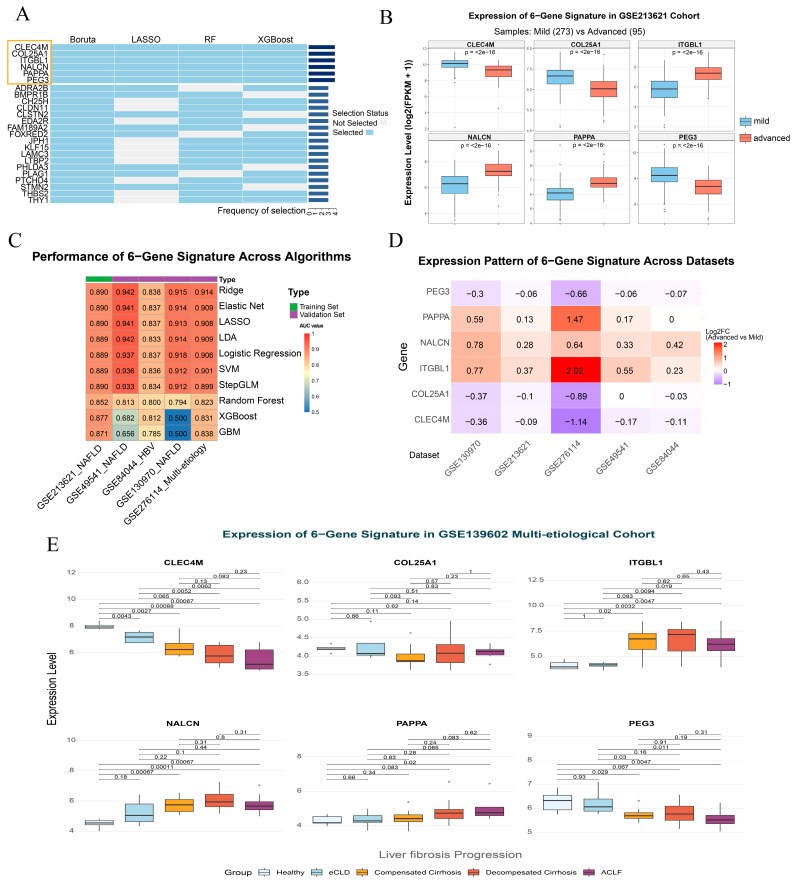
Identification, predictive performance, and cross-cohort expression reproducibility of the six-gene signature during LF progression. (**A**) Multi-algorithm consensus identifies the six core genes (CLEC4M, COL25A1, ITGBL1, NALCN, PAPPA, PEG3) consistently selected by four feature-selection methods (Boruta, LASSO, Random Forest, and XGBoost). (**B**) Differential expression of the six genes in the training cohort GSE213621 comparing mild fibrosis (F0–F2) and advanced fibrosis (F3–F4). (**C**) AUC heatmap summarizing the performance of ten machine-learning models trained on the six-gene signature, evaluated by stratified 10-fold cross-validation in the training cohort (GSE213621) and by direct external validation in four independent cohorts (GSE49541, GSE84044, GSE130970, and GSE276114). (**D**) Cross-cohort concordance of standardized expression patterns of the six genes between mild and advanced fibrosis across the four external validation cohorts, demonstrating consistent directionality of regulation across etiologies and platforms. (**E**) Stage-associated expression dynamics of the six genes across a broader clinical disease continuum in GSE139602 (healthy controls, eCLD, compensated cirrhosis, decompensated cirrhosis, and ACLF), indicating coordinated progressive shifts aligned with disease severity.

Through multi-algorithm consensus, CLEC4M, COL25A1, ITGBL1, NALCN, PAPPA, and PEG3 were jointly identified as the core six-gene signature associated with LF progression (Figure 2A). In the training cohort GSE213621, these genes displayed stage-dependent expression: CLEC4M, COL25A1, and PEG3 were significantly downregulated, whereas ITGBL1, NALCN, and PAPPA were significantly upregulated in advanced fibrosis compared with mild fibrosis (Figure 2B).

We next benchmarked ten prediction algorithms trained on the six-gene signature. Internal evaluation in GSE213621 using stratified 10-fold cross-validation showed consistently high discrimination for regularized linear models and linear classifiers, whereas ensemble methods exhibited comparatively lower performance in cross-validation (Figure 2C; Table 2). To assess generalizability, the trained models were directly applied to four independent external cohorts (GSE49541, GSE84044, GSE130970, and GSE276114). Overall, the six-gene signature maintained robust cross-cohort performance, with ridge regression achieving AUCs of 0.942, 0.838, 0.915, and 0.914 in the four validation datasets, respectively (Figure 2C; Table 2).

Importantly, robustness was supported not only by predictive accuracy but also by cross-dataset reproducibility of expression directionality. In the four external validation cohorts, heatmap analysis confirmed that the up- and down-regulation trends of all six genes between mild and advanced fibrosis were fully concordant with those observed in the training cohort (Figure 2D), supporting cross-etiology and cross-platform portability. Furthermore, in the independent cohort GSE139602 spanning a broad clinical continuum, including healthy controls, early chronic liver disease (eCLD), compensated cirrhosis, decompensated cirrhosis, and acute-on-chronic liver failure (ACLF) groups, the six genes exhibited coordinated and directionally consistent shifts along disease evolution (Figure 2E). Together, these findings indicate that this compact six-gene set not only discriminates advanced fibrosis but also tracks progressive liver disease biology.

### 2.3. Single-Cell Transcriptomics Resolves Cellular Heterogeneity and Maps the Cellular Origins of the Six-Gene Signature

**Figure 3:**
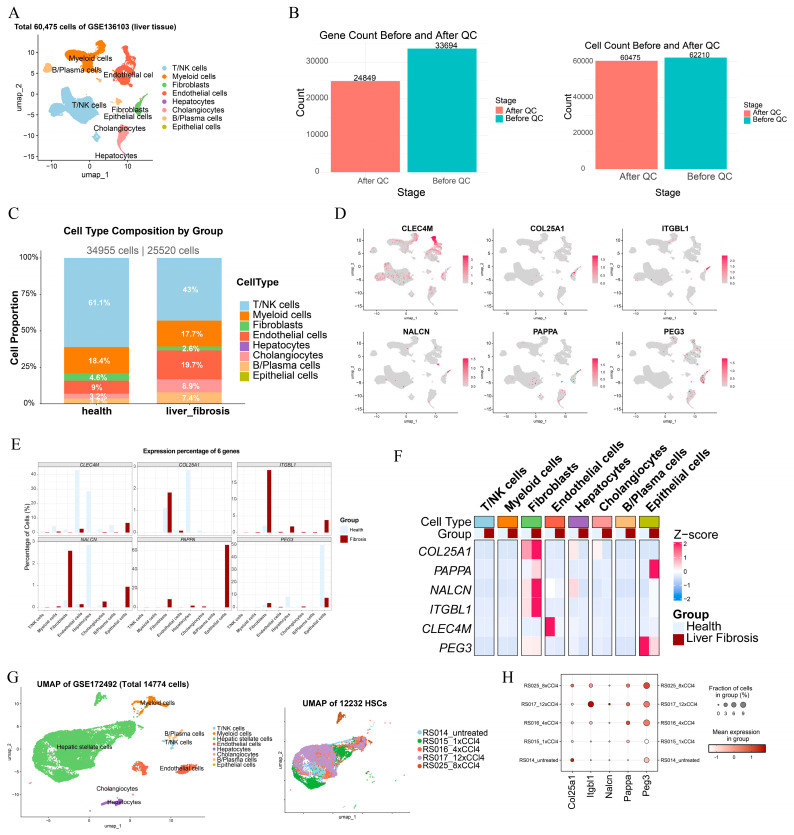
Single-cell transcriptomics resolves liver microenvironment heterogeneity and maps cellular origins of the six-gene signature. (**A**) UMAP visualization of the human liver scRNA-seq dataset GSE136103 after quality control, showing 60,475 cells from healthy controls (*n* = 5) and fibrosis patients (*n* = 5), annotated into eight major liver cell types. (**B**) Overview of single-cell quality control (QC) and filtering, showing retained cells and genes after stringent thresholds. (**C**) Cell-type composition changes between healthy and fibrotic groups in GSE136103. (**D**) Feature plots showing UMAP-level spatial expression patterns of the six signature genes (CLEC4M, COL25A1, ITGBL1, NALCN, PAPPA, PEG3). (**E**) Proportion of cells expressing each signature gene across annotated cell types. (**F**) Heatmap of Z-score-normalized mean expression of the six signature genes across the eight cell types, highlighting fibroblast enrichment for ITGBL1/NALCN/PAPPA/COL25A1, epithelial enrichment for PEG3 and endothelial enrichment for CLEC4M. (**G**) Schematic of the temporal mouse HSC fibrosis model using GSE172492, including UMAP visualization of the dataset and the HSC subset used for downstream temporal analyses. (**H**) Bubble plot showing temporal expression dynamics of five homologs (Col25a1, Itgbl1, Nalcn, Pappa, Peg3) in mouse HSCs across increasing CCl_4_ stimulation times (0, 1, 4, 8, 12). Bubble size indicates the fraction of expressing cells, and color intensity indicates mean expression.

To delineate cellular heterogeneity in the fibrotic liver microenvironment and localize the six-gene signature at single-cell resolution, we analyzed the human liver single-cell RNA sequencing (scRNA-seq) dataset GSE136103, which includes liver tissue from 5 patients with fibrosis and 5 healthy controls. After stringent quality control, 60,475 high-quality cells (34,955 healthy; 25,520 fibrotic) and 24,849 genes were retained for downstream analyses. Dimensionality reduction, unsupervised clustering, and annotation using canonical marker genes identified eight major liver cell populations: T/NK cells, myeloid cells, endothelial cells, cholangiocytes, B/plasma cells, fibroblasts, epithelial cells, and hepatocytes (Figure 3A,B).

Cellular composition analysis indicated substantial microenvironmental remodeling in fibrosis (Figure 3C). In the fibrotic group, the fraction of endothelial cells increased from 8.97% to 19.71%, cholangiocytes increased from 3.16% to 8.94%, and B/plasma cells increased from 3.66% to 7.44%. In contrast, T/NK cells decreased from 61.09% to 43.02%, whereas myeloid cells remained relatively stable (18.43% vs. 17.69%). The relative proportion of fibroblasts was lower in fibrosis (4.59% vs. 2.57%), noting that cell-type proportions in scRNA-seq may be influenced by tissue dissociation and capture efficiencies. Collectively, these shifts are consistent with prominent vascular remodeling, ductular reaction, and immune microenvironment changes during fibrogenesis.

We next profiled the expression landscape of the six signature genes (CLEC4M, COL25A1, ITGBL1, NALCN, PAPPA, PEG3) across cell types (Figure 3D–F). At the aggregated level, ITGBL1, NALCN, and PAPPA showed increased expression in fibrosis, whereas CLEC4M was markedly downregulated; COL25A1 and PEG3 did not show clear global differences between groups. Importantly, cell-type-resolved analyses revealed distinct cellular origins. ITGBL1, NALCN, and PAPPA were predominantly enriched in fibroblasts, with the strongest signals observed in fibrotic fibroblasts (mean expression: ITGBL1 = 0.7519, NALCN = 0.0371, PAPPA = 0.2355), consistent with the role of fibroblast-lineage cells as principal effectors of extracellular matrix remodeling. In contrast, CLEC4M was highly expressed in endothelial cells in the healthy state (mean expression 5.6959, Z-score = 2.00) but decreased sharply in fibrotic endothelial cells (0.1878), occurring alongside expansion of the endothelial compartment. This pattern suggests an endothelial state shift during fibrosis, characterized by population expansion accompanied by transcriptional reprogramming. Additionally, PAPPA showed relative enrichment in epithelial cells, while PEG3 exhibited a more heterogeneous distribution across subsets. Together, these data provide a cell-context framework for interpreting the six-gene signature, linking it primarily to fibroblast activation and endothelial remodeling.

Further cell-type-resolved analysis revealed distinct shifts in the cellular distribution of the six genes during fibrosis (Table 3). In healthy liver, COL25A1 expression was broadly distributed, with 51.5% originating from fibroblasts, 24.2% from hepatocytes, and 20.0% from cholangiocytes. In fibrosis, however, COL25A1 became almost exclusively restricted to fibroblasts (98.6% of its total expression), while its expression was lost in other cell types. This increased fibroblast contribution occurred despite a reduction in the relative abundance of fibroblasts in fibrotic samples (from 4.59% to 2.57%), explaining the decrease in COL25A1 expression observed at the bulk tissue level. NALCN showed a marked shift toward fibroblast enrichment: its fibroblast-derived fraction increased from 33.6% in health to 81.8% in fibrosis. PEG3 was predominantly expressed in epithelial cells in healthy liver (79.3% of total expression), with a smaller fibroblast contribution (11.2%). In fibrosis, the epithelial fraction decreased to 51.4%, while the fibroblast fraction increased to 38.8%, consistent with its overall downregulation in bulk tissue. PAPPA, in addition to its fibroblast enrichment (80.7% in health, 15.3% in fibrosis), showed negligible expression in healthy epithelial cells but became markedly induced in fibrotic epithelium, where it accounted for 83.2% of its total expression. The cell-type-specific expression distributions of the six genes are further illustrated in Supplementary Figures S7–S13. Together, these data provide a cellular context for interpreting the six-gene signature, linking it primarily to fibroblast activation and endothelial remodeling, with additional contributions from epithelial cell populations.

Given that HSCs are the principal ECM-producing population in the liver [26], we further performed validation using a temporal mouse scRNA-seq dataset, GSE172492 (Figure 3G). This dataset models progressive fibrotic stimulation through repeated CCl_4_ exposures (0, 1, 4, 8, 12 times). The expression proportion analyses demonstrated coordinated regulation of five detectable homologs in HSCs (Figure 3H): Itgbl1, Nalcn, and Pappa increased progressively with stimulation, whereas Col25a1 decreased, consistent with the directionality observed in human tissues. Peg3 showed a dynamic trajectory, with early downregulation followed by later upregulation. These findings support that key components of the signature are actively regulated in the major fibrogenic effector cell population and are conserved during fibrotic progression.

### 2.4. Drug Repositioning Prediction via CMap Identifies Potential Therapeutic Compounds for LF

**Figure 4:**
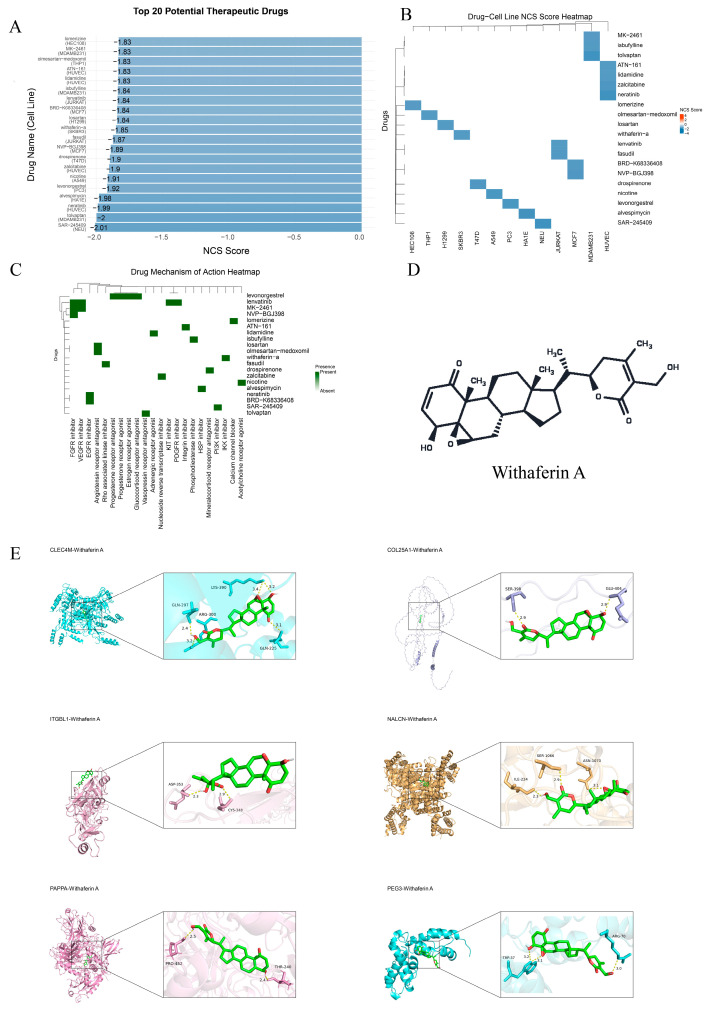
CMap-based drug repositioning and molecular docking nominate candidate anti-fibrotic compounds and prioritize WFA. (**A**) CMap-based ranking of candidate compounds by NCS. The bar chart shows the top 20 compounds with significant inverse connectivity to the fibrosis-associated signature (FDR < 0.05), ordered from most negative to least negative NCS. More negative NCS values indicate stronger predicted antagonism of the fibrotic transcriptional program. (**B**) Compound–cell line NCS response matrix. Heatmap of NCS values for the top 20 compounds across multiple CMap reference cell lines/conditions, illustrating heterogeneity of connectivity signals across cellular contexts and supporting context-dependent activity. (**C**) MOA landscape of top candidates. Heatmap summarizing annotated MOAs for the top 20 compounds, including PI3K/EGFR/FGFR inhibition, vasopressin receptor antagonism, HSP inhibition, and angiotensin receptor antagonism. (**D**) Chemical structure of WFA, selected for downstream experimental validation. (**E**) Molecular docking of WFA against proteins encoded by the six-gene signature. Representative docking poses illustrate key residue-level interactions for selected targets.

To identify potential therapeutics capable of counteracting the transcriptional program associated with the six-gene LF signature, we performed reverse signature matching using the CMap. Compounds with significant connectivity (FDR < 0.05) and well-annotated mechanism of action (MOA) were retained and ranked by normalized connectivity score (NCS). We prioritized visualization of the top 20 compounds with the most negative NCS values (NCS range: −2.007 to −1.829) (Figure 4A). Negative NCS values indicate an inverse correlation between drug-induced perturbation profiles and the fibrosis-associated signature, suggesting a potential to antagonize the disease-related transcriptional state. The highest-ranked compound was SAR-245409 (NCS = −2.007), a PI3K inhibitor showing its strongest negative connectivity in the NEU cell line. The next highest-ranked candidates included tolvaptan (NCS = −1.997, vasopressin receptor antagonist) and neratinib (NCS = −1.988, EGFR inhibitor) (Figure 4A).

To evaluate context dependence of candidate responses, we constructed a compound–cell line NCS matrix (Figure 4B). The heatmap revealed pronounced heterogeneity of connectivity signals across cellular contexts, indicating that the predicted “reversal” effects are likely cell-state and lineage dependent. For example, SAR-245409 exhibited its strongest negative connectivity in NEU, whereas tolvaptan showed more negative connectivity in MDAMB231, highlighting potential microenvironmental or lineage constraints on transcriptional reversal (Figure 4B).

We next summarized the MOA distribution of the top 20 candidates (Figure 4C). These compounds collectively mapped to pharmacological classes relevant to inflammation, proliferation, and microenvironment remodeling, including PI3K inhibition, EGFR inhibition, FGFR inhibition (e.g., NVP-BGJ398, lenvatinib, MK-2461), vasopressin receptor antagonism (tolvaptan), HSP inhibition (avespimycin), and angiotensin receptor antagonism (losartan, olmesartan medoxomil). Notably, lenvatinib exhibited multi-target inhibitory properties (FGFR/KIT/PDGFR/VEGFR), suggesting a potential capacity to influence broader fibrosis-associated signaling networks. The top 20 candidate compounds ranked by NCS are listed in Supplementary Table S1.

Considering drug availability, known safety information, and prior evidence supporting anti-inflammatory/antioxidant activities relevant to fibrosis, we selected Withaferin A (WFA) for subsequent experimental validation [27,28] (Figure 4D). To further explore potential target engagement at the protein level, we performed in silico molecular docking between WFA and proteins encoded by the six core genes. Docking results indicated that NALCN yielded the most favorable predicted binding affinity (−9.9 kcal/mol) (Figure 4E; Table 4). The top-ranked NALCN–WFA pose featured a hydrogen bond with ILE-224 (2.3 Å), supported by additional contacts involving SER-1066 and ASN-1070. These docking results suggest NALCN as a prioritized candidate for target-engagement validation.

### 2.5. WFA Significantly Ameliorates CCl_4_-Induced LF in Mice and Is Associated with Broad Transcriptomic Reversal of Fibrotic Programs

**Figure 5:**
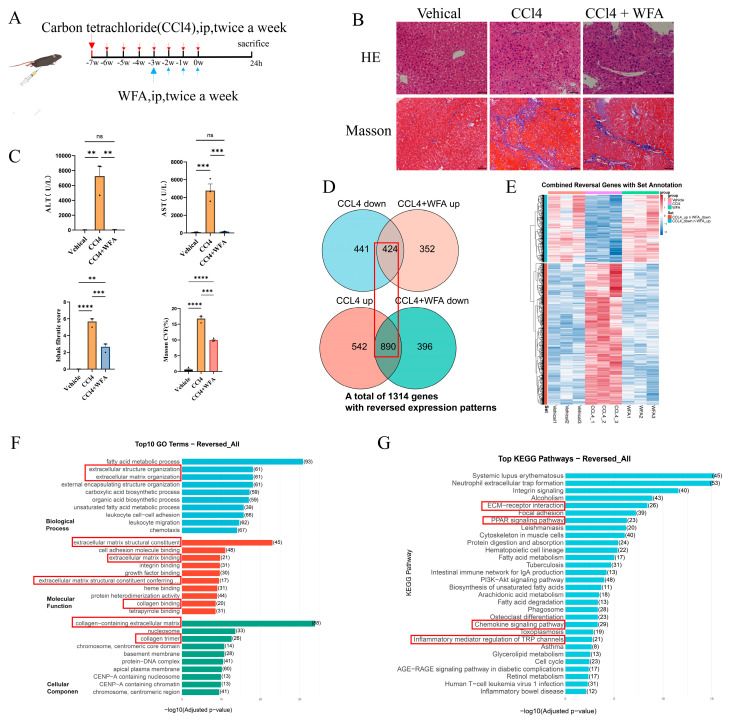
Withaferin A ameliorates CCl_4_-induced LF in mice and is associated with transcriptomic reversal of fibrotic programs. (**A**) Schematic of the CCl_4_-induced mouse LF model and WFA intervention protocol. Liver tissues and sera were collected 24 h after the final administration for histology, biochemistry, and transcriptomic analyses. (**B**) Representative histological assessment of liver injury and collagen deposition. Upper: H&E staining showing reduced hepatocellular injury and inflammatory infiltration following WFA treatment. Lower: Masson’s trichrome staining demonstrating decreased collagen deposition in the WFA group (blue staining indicates collagen; scale bar, 50 μm). (**C**) Quantification of liver injury and fibrosis. Serum aspartate aminotransferase (AST) and alanine aminotransferase (ALT) levels, Ishak fibrosis scores, and collagen volume fraction (CVF) derived from Masson-stained sections are shown. Data are presented as mean ± SEM. Statistical significance: ns, not significant, ** *p* < 0.01, *** *p* < 0.001, **** *p* < 0.0001. (*n* = 3 per group). (**D**) Identification of WFA-associated “reversal genes.” Venn diagram showing the overlap between differentially expressed genes (DEGs) in “CCl_4_ vs. Vehicle” and “CCl_4_ + WFA vs. CCl_4_.” Genes upregulated in fibrosis and downregulated by WFA (*n* = 890), together with genes downregulated in fibrosis and upregulated by WFA (*n* = 424), constitute 1314 reversal genes that exhibit opposite directional changes between the two comparisons. (**E**) Heatmap showing the expression patterns of the 1314 reversal genes across the three experimental groups (Vehicle, CCl_4_, CCl_4_ + WFA). (**F**) GO biological process enrichment analysis of the 1314 reversal genes. (**G**) KEGG pathway enrichment analysis of the 1314 reversal genes.

The candidate drug WFA demonstrated significant therapeutic efficacy in a CCl_4_-induced mouse model of LF. As outlined in the experimental design (Figure 5A), WFA treatment markedly alleviated histological damage, reducing hepatocyte necrosis and inflammatory infiltration. Consistent with this, Masson’s trichrome staining revealed a substantial decrease in collagen deposition (*p* < 0.05) and fibrosis staging scores (*p* < 0.05), confirming its inhibition of pathological ECM accumulation. In parallel, serum levels of ALT (*p* < 0.05) and AST (*p* < 0.05) were significantly lower in the WFA-treated group, indicating restored liver function (Figure 5B,C). These results collectively establish the anti-fibrotic effects of WFA at both tissue-structural and functional levels.

To gain mechanistic insights, we performed bulk RNA-seq of liver tissues. To identify the genes whose expression changes induced by CCl_4_ were reversed by WFA treatment, the genes upregulated after CCl_4_ intervention were intersected with the genes downregulated after WFA treatment, and the genes downregulated after CCl_4_ intervention were intersected with the genes upregulated after WFA treatment (Figure 5D). A total of 1314 genes with opposite expression patterns were identified and defined as WFA reversal genes. The expression patterns of these reversal genes across the groups are shown in the heatmap (Figure 5E). These genes were altered after CCl_4_ intervention and were restored to the same expression patterns as the control group following WFA treatment. The GO enrichment analysis results show that these reversal genes are primarily involved in extracellular matrix (ECM) organization and structural remodeling, as well as collagen formation, which are key pathological mechanisms in liver fibrosis (Figure 5F). The KEGG pathway analysis further revealed enrichment in important pathways reported to regulate liver fibrosis progression, including ECM−receptor interaction, inflammatory mediator regulation of TRP channels, PPAR signaling, and the chemokine signaling pathway (Figure 5G). Together, these transcriptomic findings suggest that WFA attenuates LF through multi-axis remodeling involving ECM dynamics, metabolic reprogramming, and inflammatory–immune responses.

### 2.6. WFA Suppresses TGF-β1-Induced Activation of Human HSC Cell Line and Reduces ECM Production In Vitro

**Figure 6:**
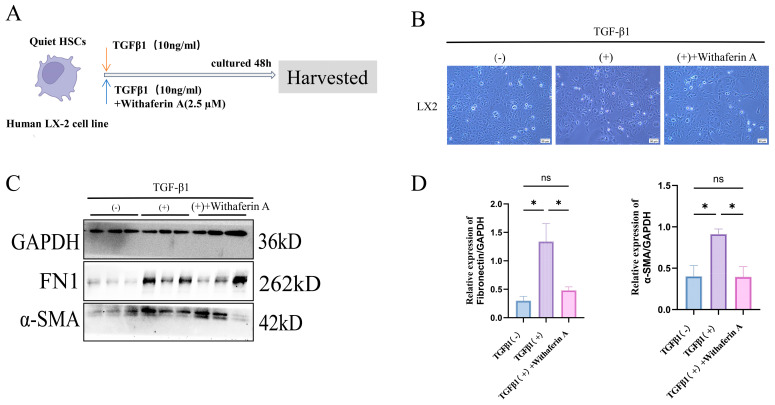
WFA inhibits TGF-β1-induced activation of HSCs and ECM production in vitro. (**A**) Schematic of the LX-2 cell treatment groups: Control, TGF-β1 (10 ng/mL), and TGF-β1 + WFA (2.5 μM). Cells were harvested after 48 h for downstream analysis. (**B**) Representative phase-contrast images showing treatment-associated morphological changes in LX-2 cells. (**C**) Representative Western blotting images for α-SMA and FN1. (**D**) Densitometric quantification of α-SMA and FN1 normalized to GAPDH. Data are presented as mean ± SEM. (ns, not significant, * *p* < 0.05).

To investigate whether WFA exerts a direct anti-fibrotic effect on HSCs, we used an in vitro activation model in the human HSC cell line LX-2. Cells were stimulated with TGF-β1 (10 ng/mL) in the presence or absence of WFA (2.5 μM) according to the experimental scheme (Figure 6A). Consistent with HSC activation, TGF-β1 induced morphological changes characteristic of an activated phenotype, especially cellular edema (Figure 6B). We next assessed canonical fibrogenic markers by Western blotting, including α-SMA as an indicator of HSC activation and FN1 as a major ECM component. TGF-β1 stimulation markedly increased α-SMA and FN1 protein abundance compared with controls. Notably, co-treatment with WFA substantially attenuated these TGF-β1-induced increases, reducing the levels of both α-SMA and FN1 (Figure 6C,D). Together, these data indicate that WFA directly counteracts HSC activation and ECM production in response to TGF-β1, providing mechanistic support for the anti-fibrotic effects observed in vivo.

## 3. Discussion

LF represents a dysregulated healing response to chronic liver injury, characterized by excessive deposition of ECM, which ultimately leads to organ dysfunction and can promote malignant transformation [29,30]. While etiology-specific treatments (such as antiviral therapy, alcohol abstinence, or weight loss) can slow disease progression [18,30], effective drugs that directly reverse or halt fibrotic advancement remain critically lacking [30,31]. This highlights an urgent need for etiology-agnostic, robust biomarkers and novel therapeutic targets.

Confronting LF—a highly heterogeneous process driven by multicellular interactions—traditional research strategies relying on single algorithms or cohorts often yield results with limited robustness and generalizability [32,33,34,35]. For instance, PNPLA3-based signatures identified in NAFLD cohorts [36,37] or markers like serum Golgi protein 73 levels identified in HBV cohorts [34], while effective within their specific contexts, often face challenges in generalizing to real-world patients with mixed etiologies. To systematically mitigate biases from algorithmic selection and single-etiology specificity, this study integrated multi-etiology training and external validation sets, employing a consensus screening approach with four complementary machine learning algorithms.

The final six-gene signature maintained an average AUC of 0.864 across four independent cohorts encompassing NAFLD, HBV, and mixed etiologies. Compared to recent efforts in developing pan-etiology biomarkers [29,38], such as those based on cellular senescence or inflammation-related gene sets, our signature achieves comparable accuracy with a markedly smaller gene set, supporting its potential practical value for classification and translational deployment.

The scRNA-seq analysis unveiled the evolving LF microenvironment. Utilizing datasets GSE136103 and GSE172492, we delineated shifts in cellular composition, notably observing a significant increase in the proportions of endothelial and cholangiocyte cells in fibrosis. Beyond composition, the six-gene signature was anchored to specific cell populations, providing a cellular context for its diagnostic signal. COL25A1, ITGBL1, and NALCN were predominantly enriched in activated fibroblasts. While COL25A1 has been predominantly studied in neurological contexts [39,40,41] and skin wound healing [42], our study extends its relevance to LF and further suggests divergent expression trends between human tissue and a temporal mouse stellate-cell model, implying context-dependent regulation. NALCN, a sodium leak channel [43,44], was enriched in activated fibroblasts, raising the possibility that ion homeostasis programs may participate in fibroblast activation states. ITGBL1 has been implicated in matrix remodeling and pro-fibrotic programs [45,46,47] and can promote fibrotic stroma formation in pancreatic cancer via integrin signaling [48]. Our results refine this literature by pinpointing its major cellular source in fibrotic liver tissue and supporting ITGBL1 as a conserved pro-fibrotic node. For CLEC4M, previous studies have reported context-dependent roles in oncology: some indicate that CLEC4M can inhibit the progression of HCC and is associated with a favorable prognosis [49], while others suggest that high levels of CLEC4M correlate with a poor prognosis in HCC patients [50]. Here, CLEC4M was most prominent in healthy endothelial cells and was markedly downregulated in fibrotic samples. Coupled with endothelial cell expansion, this pattern is consistent with endothelial remodeling during fibrogenesis and supports the concept that LSEC dysfunction and “capillarization” are key events shaping the fibrotic microenvironment. PEG3 is a transcription factor that exhibits tumor-suppressive effects in certain cancers, and its mutation may lead to a poor prognosis in some tumors [51,52,53]. PEG3 appears to present contradictory manifestations in LF: some studies indicate that PEG3 expression is upregulated in activated HSCs during NASH-induced LF [54], while other research indicates that PEG3 is a target gene of miR-129-5p and alleviates CCl_4_-induced LF in rats by inhibiting the NF-κB signaling pathway [55]. PAPPA, primarily originating from epithelial and fibroblast cells and known for its role in the reproductive system [56], has recently been linked to adipose tissue remodeling [57]. Taken together, these findings map the cellular origins and plausible functional axes of the six-gene signature and support its biological relevance beyond purely statistical selection.

On the translational front, this study treated the high-consensus gene expression signature as a disease “molecular phenotype”, leveraging CMap for reverse drug matching to extend biomarker discovery into therapeutic hypothesis generation. Among the candidate compounds, WFA, a natural product with documented anti-inflammatory and antioxidant properties [58,59,60], was selected for in-depth validation. Although WFA has shown protective effects in drug- and alcohol-induced liver injury [61,62,63], its anti-fibrotic efficacy and mechanistic footprint in well-established fibrogenic settings have not been fully characterized. Here, we provide systematic evidence that WFA alleviates fibrotic injury in a CCl_4_-induced mouse model. This aligns with previous findings demonstrating the anti-fibrotic effect of WFA in reversing bile duct ligation (BDL)-induced LF [64]. We demonstrate that WFA confers protection at both histological and functional levels. Importantly, we further extend these in vivo observations to a human HSC model by showing that WFA directly attenuates TGF-β1-induced activation of LX-2 cells and suppresses ECM production. Further hepatic bulk RNA-seq results revealed that WFA specifically reversed 1314 fibrosis-related DEGs. These genes were significantly enriched in modules related to fatty acid metabolism, ECM organization, focal adhesion, and PPAR signaling pathways, suggesting that WFA’s efficacy may stem from coordinated modulation of metabolic dysregulation and ECM dynamics.

This study has several limitations. First, despite multi-cohort validation, the heterogeneity inherent in retrospective public datasets necessitates further evaluation of diagnostic performance in prospective, multi-center clinical cohorts. Second, our experimental validation used a single in vivo dosing regimen and a single in vitro concentration window. Furthermore, the CCl_4_ model induces LF via hepatotoxic injury. Although WFA attenuated fibrosis and reversed its transcriptome, this model cannot distinguish a direct anti-fibrotic effect from an indirect hepatoprotective one. Future studies using a treatment-reversal protocol or a diet-induced NASH model would better delineate WFA’s mechanism in an etiology relevant to the NAFLD-derived signature. In addition, we did not perform an acute toxicity test to select the WFA dose; formal toxicity and comprehensive dose–response studies would be necessary to establish optimal efficacy and safety profiles and strengthen dose selection in future work. Future studies with larger animal sample sizes, broader dose ranges, and multiple time points are needed to fully characterize the dose–response relationship and temporal dynamics of WFA’s anti-fibrotic effects. The in vitro assessment of HSC deactivation was limited to α-SMA and Fibronectin. A more complete picture would require assays for proliferation, contractility, apoptosis, and cell viability to exclude cytotoxicity. Third, while we demonstrated robust phenotypic efficacy and transcriptome-level pathway reversal, mechanistic interrogation largely remained at the level of marker proteins and enrichment analyses. The docking results should be viewed as hypothesis-generating. The predicted target engagement (e.g., NALCN) was not directly validated experimentally, and subsequent functional experiments did not explicitly test these candidate protein interactions. Future work should therefore incorporate causal validation strategies—such as genetic knockdown or overexpression, pathway reporters, and orthogonal target-engagement assays—to determine whether and how specific predicted targets mediate WFA’s downstream signaling effects. Additionally, the consensus gene signature used for drug prediction was not necessarily expected to be fully reversed as an aggregate in bulk tissue, given the cellular complexity of the liver and the cell-type specificity revealed by scRNA-seq. Cell-resolved signatures and deconvolution-aware drug matching may further improve precision in subsequent studies.

## 4. Materials and Methods

### 4.1. Data Acquisition

This study included eight publicly available LF-related transcriptomic datasets retrieved from the Gene Expression Omnibus (GEO) database [65], comprising six bulk transcriptome datasets and two scRNA-seq datasets (Table 5). For bulk datasets, we performed uniform preprocessing procedures, including gene annotation, removal of duplicated gene symbols, sample curation and matching based on clinical metadata, and expression normalization. Based on clinical fibrosis staging, samples were recategorized into a binary outcome: mild fibrosis (F0–F2) versus advanced fibrosis (F3–F4). For scRNA-seq analyses, GSE136103 (human) was used to characterize cell-type-specific expression patterns of the signature genes, whereas GSE172492 (mouse) was used to validate their expression changes in a temporal fibrosis model.

### 4.2. Machine Learning

During feature selection, four complementary machine-learning algorithms were applied in parallel to the training NAFLD cohort GSE213621 to reduce reliance on any single method and improve selection robustness. Preprocessing included quantile normalization and filtering of lowly expressed genes; specifically, genes with expression levels below the 25th percentile across all samples were removed. Based on fibrosis stage, samples were formulated as a binary classification task: mild fibrosis (F0–F2) versus advanced fibrosis (F3–F4). The algorithms used were: (1) Boruta, a random forest-based all-relevant feature selection approach that identifies stable associations by comparing original variables with permuted “shadow features”; (2) LASSO regression, which applies L1 regularization to obtain a sparse subset of discriminative genes; (3) Random Forest, which ranks feature importance using out-of-bag performance and impurity-based metrics; and (4) XGBoost, which quantifies feature contribution using gain, cover, and frequency [68]. Genes selected by at least two algorithms were defined as high-consensus genes, and genes consistently selected by all four algorithms were retained as the final core features.

For model construction and evaluation, prediction models based on the core features were trained using ten algorithms spanning regularized linear models, traditional statistical models, linear classifiers, and ensemble learning methods. Model performance was primarily evaluated using the area under the receiver operating characteristic curve (AUC). Internal validation was performed using stratified 10-fold cross-validation within GSE213621 to reduce overfitting. For external validation, finalized models were applied without re-training to four independent cohorts (GSE49541, GSE84044, GSE130970, and GSE276114) to assess cross-etiology and cross-cohort generalizability. In addition, GSE136092 was used to examine expression dynamics across fibrosis progression. All comparisons were conducted using consistent outcome definitions, and model hyperparameters were optimized via cross-validation in the training cohort to ensure comparability across algorithms.

### 4.3. scRNA-Seq Analysis

The two scRNA-seq datasets, GSE136103 and GSE172492, were processed in R (v4.4.1) using Seurat (v4) [69]. Raw gene–cell count matrices were subjected to quality control using a multi-metric filtering strategy to remove low-quality cells. Specifically, only cells meeting all of the following criteria were retained: 300 ≤ nFeature_RNA ≤ 7000, percent.mt < 20%, percent.hb < 3%, and nCount_RNA < 100,000. Genes detected in fewer than three cells were excluded to reduce noise from low-abundance transcripts. After quality control, data were normalized and variance-stabilized using SCTransform [70]. Dimensionality reduction was performed by principal component analysis (PCA). The first 20 principal components (dims = 1:20) were used to construct the shared nearest neighbor (SNN) graph (FindNeighbors) and for downstream clustering (FindClusters) with a resolution of 0.5. Cells were visualized in two dimensions using Uniform Manifold Approximation and Projection (UMAP) based on the same principal components (RunUMAP; dims = 1:20). Major clusters were annotated into canonical liver cell types based on established marker genes. The selection of quality-control thresholds—particularly for mitochondrial gene content—and the choice of dimensionality parameters were informed by recommendations emphasizing tissue- and cell type-specific adaptations [71]. Quality-control visualization, UMAP embedding, and cluster marker annotation for GSE136103 are provided in Supplementary Figures S1–S4. The corresponding UMAP embedding and cluster marker annotation for GSE172492 are shown in Supplementary Figures S5 and S6.

### 4.4. CMap Analysis and Drug Repositioning

Drug screening was performed using the CMap resource accessed via the CLUE platform (https://clue.io/, accessed in 10 October 2025), which quantifies associations between disease molecular signatures and small-molecule perturbagens based on gene expression profiles [72]. The high-consensus gene set identified from the training cohort was used to construct the disease signature. Specifically, genes were stratified into up-regulated and down-regulated lists according to their differential expression in the advanced fibrosis group relative to the mild fibrosis group. These gene lists were submitted to CMap as query signatures. For each compound across multiple cell lines/conditions, CMap returned an NCS and the corresponding FDR q-value. Candidate compounds were prioritized using the following criteria: (1) only significant results were retained (FDR < 0.05); (2) only entries with explicit annotations (e.g., compound identity and mechanism-of-action metadata) were included; and (3) compounds were ranked by NCS from the most negative to the least negative. A negative NCS indicates that the drug-induced transcriptional perturbation is inversely correlated with the disease signature, suggesting potential to reverse the disease-associated expression program [72,73]. Based on these criteria, the top 20 small molecules with the lowest NCS were selected for downstream analyses and visualization. To evaluate the consistency of candidate effects across cellular contexts and to summarize potential pharmacological mechanisms, we generated a heatmap of the compound–cell line NCS matrix and an annotation plot summarizing MOA, thereby highlighting shared activity patterns and clustering features among prioritized compounds [74].

### 4.5. Molecular Docking

The three-dimensional structures of the target proteins were retrieved via UniProt and the corresponding structural resources linked to each UniProt entry (https://www.uniprot.org/, accessed in 10 October 2025) [75,76]. To evaluate the potential interactions between the candidate drug and the proteins encoded by the six core genes, molecular docking was performed in a multi-target screening manner. The candidate compound was selected from CMap prioritization results as described above. Prior to docking, protein structures were prepared using AutoDockTools (v1.5.7) by removing crystallographic water molecules, adding polar hydrogen atoms, assigning Gasteiger charges, and merging non-polar hydrogens. The processed receptor structures were saved in PDBQT format for downstream docking [77]. For ligand preparation, the three-dimensional structure of the candidate drug was generated and geometry-optimized using ChemDraw 19.0 [78]. Docking was subsequently carried out using AutoDock Vina (v1.2.3) under a semi-flexible docking setting (rigid receptor and flexible ligand) to predict binding poses and binding affinities (Vina scores, kcal/mol) between the candidate drug and each receptor. The top-ranked pose for each target was retained for interaction analysis, and key residue-level interactions (e.g., hydrogen bonds and supporting contacts) were visualized and inspected using PyMOL (v2.5.0) [79].

### 4.6. Experimental Animals

Fifteen 4-week-old male C57BL/6J mice (20–25 g) were purchased from GemPharmatech Co., Ltd. (Nanjing, China). After one week of acclimatization, mice were randomly assigned to three groups (*n* = 5 per group): a corn oil vehicle control group, an LF model group, and a WFA treatment group. All animal procedures were conducted in accordance with the National Institutes of Health Guide for the Care and Use of Laboratory Animals and were approved by the Institutional Animal Care and Use Committee. To establish the LF model, mice in the fibrosis model and WFA treatment groups received intraperitoneal (i.p.) injections of 25% (*v*/*v*) CCl_4_ (Macklin, Shanghai, China, C805325) in corn oil (Abmole, Houston, TX, USA, M9109) at 5 μL/g body weight, twice weekly for 8 weeks. Control mice received i.p. injections of an equal volume of corn oil on the same schedule. To evaluate the curative effect of WFA on established fibrosis, beginning at week 5, mice in the WFA treatment group additionally received i.p. injections of WFA (TargetMol, Boston, MA, USA, T5687) at 5 mg/kg, prepared in 5% dimethyl sulfoxide (DMSO) in corn oil (5:95, *v*/*v*) and administered in parallel with the CCl_4_ injections. The WFA dose was selected based on previous studies [80]. Twenty-four hours after the final injection, mice were fasted and anesthetized with isoflurane (5% for induction, 2% for maintenance). Whole blood was collected via retro-orbital bleeding under surgical anesthesia, allowed to clot at room temperature for 30 min, and centrifuged at 3000× *g* for 15 min at 4 °C to obtain serum for biochemical analyses. Immediately after blood collection, mice were deeply anesthetized with 5% isoflurane until respiratory arrest, followed by cervical dislocation to ensure euthanasia. Livers were rapidly excised; portions of tissue were snap-frozen in liquid nitrogen and stored at −80 °C for subsequent molecular analyses.

### 4.7. Histopathological Assessment of Liver Tissues

Liver tissues were fixed, paraffin-embedded, and sectioned at 4 μm. Sections were stained with hematoxylin and eosin (H&E) and Masson’s trichrome to assess histopathological changes and collagen deposition, respectively. Fibrosis stage, inflammatory activity and necrotic activity were semi-quantitatively evaluated by two independent pathologists under double-blind conditions using the Ishak fibrosis scoring system [81]. For quantitative analysis of collagen deposition, Masson’s trichrome-stained sections were analyzed with ImageJ 1.8.0 (NIH, Bethesda, MD, USA) [82,83]. For each sample, five randomly selected, non-overlapping medium- to high-power fields were captured. The collagen-positive area (blue staining) was measured and expressed as a percentage of the total field area, and the mean value across the five fields was calculated for each sample. All liver sections were taken from the left lateral lobe at the same anatomical location to ensure consistency.

### 4.8. Serum Biochemical Analysis

To evaluate liver function, serum AST and ALT levels were measured using an automated biochemical analyzer (Mindray, Shenzhen, China, BS-360S) at Chengdu Aochuang Biotechnology Co., Ltd. (Chengdu, China). The assays were performed using an ALT kit (Mindray, 105-000442-00) and an AST kit (Mindray, 105-000443-00). The detection limits were as specified by the manufacturer for this instrument.

### 4.9. RNA Sequencing and Bioinformatic Analysis

Total RNA was extracted from snap-frozen mouse liver tissues using TRIzol reagent (Invitrogen, Thermo Fisher Scientific, Waltham, MA, USA). After quality assessment, sequencing libraries were constructed using the Hieff NGS^®^ Ultima Dual-mode mRNA Library Prep Kit (Yeasen Biotechnology, Biotechnology, Shanghai, China), and paired-end sequencing was performed on an Illumina HiSeq™ 4000 platform. Sequencing was conducted by Guangzhou Genedenovo Biotechnology Co., Ltd. (Guangzhou, China). Raw sequencing data underwent quality control (e.g., FastQC [84]) and normalization. Transcripts per million (TPM) expression matrices were generated after normalization for sequencing depth and gene/transcript length. Genes with zero expression across all samples were excluded. The processed expression matrix is provided in the Supplementary Table. Differentially expressed genes (DEGs) were identified using an adjusted *p* value < 0.05 (Benjamini–Hochberg procedure) and an absolute fold change (FC) ≥ 1.5. Data visualization and pathway enrichment analyses were performed in R. Heatmaps were generated using the pheatmap and ggplot2 packages. GO and KEGG enrichment analyses were conducted using the clusterProfiler package.

### 4.10. Cell Culture

The human HSC cell line LX-2 (STCC10105, Zishan Biological Company, Shanghai, China) was cultured in complete growth medium consisting of DMEM-F12 (Gibco, Thermo Fisher Scientific, C11330500BT) supplemented with 10% fetal bovine serum (FBS; Cellmax, Beijing, China, SA201.02) and 1% penicillin-streptomycin (Biosharp, Hefei, China, BL505A) at 37 °C in a humidified atmosphere with 5% CO_2_. For cell stimulation, recombinant TGF-β1 (Peprotech, Rocky Hill, NJ, USA, 100-21) was reconstituted and diluted in the complete medium to a final working concentration of 10 ng/mL. For drug treatment, WFA was first dissolved in DMSO to prepare a 10 mM stock solution, which was then diluted in the complete medium to reach a final working concentration of 2.5 µM [85]. The final concentration of DMSO in the medium was maintained at 0.1% (*v*/*v*) or lower, and an equal volume of DMSO (0.1%) was added to the control and TGF-β1-stimulated groups as a vehicle control.

### 4.11. Western Blot Analysis

Total cellular proteins were extracted and quantified. Protein samples (20 µg per lane) were mixed with loading buffer, denatured by boiling, and separated by sodium dodecyl sulfate–polyacrylamide gel electrophoresis (SDS-PAGE). Subsequently, the separated proteins were transferred onto a polyvinylidene fluoride (PVDF) membrane (Millipore, Burlington, MA, USA). After transfer, the membrane was blocked with 5% skim milk for 1 h at room temperature. It was then incubated overnight at 4 °C with the following primary antibodies: anti-Fibronectin (FN1, Abcam, Cambridge, UK, ab2413; 1:1000) and anti-α-SMA (CST, Danvers, MA, USA, 19245S; 1:1000). Following washes, the membrane was incubated with the corresponding fluorescent secondary antibody (CST, 5151) for 1 h at room temperature under light-protected conditions. Protein bands were visualized and captured using a BioRad imaging system. GAPDH (Abclonal, Wuhan, China, A19056; 1:5000) was used as the loading control, and the band intensities were quantified using ImageJ 1.8.0 software.

### 4.12. Statistical Analysis

All bioinformatics analyses were performed using R software (version 4.4.1). Continuous variables are presented as the mean ± standard error of the mean (SEM). One-way analysis of variance (ANOVA) was performed to compare three or more groups, followed by Bonferroni’s post hoc test. Statistical significance was set at *p* value < 0.05. Statistical graphs were generated using GraphPad Prism software (version 10.0).

## 5. Conclusions

In conclusion, this study identified a robust, cross-etiology six-gene signature for LF progression through multi-algorithm consensus machine learning, delineated its cellular origins using single-cell transcriptomics, and predicted the promising therapeutic candidate WFA. Preliminary mechanisms of its action were supported by in vivo and in vitro experiments. This work not only provides novel potential diagnostic biomarkers and a therapeutic candidate for LF but also lays a foundation for subsequent target validation and the development of precise anti-fibrotic strategies.

## Figures and Tables

**Table 1:** Intersection analysis of feature selection by four machine learning algorithms.

Algorithm	Number of Selected Genes/Features	Key Parameters/Metrics
Boruta	65	Importance score (Z-score) > shadow features
LASSO	33	λ = lambda.1se (selected via 10-fold cross-validation)
Random Forest	100	Mean Decrease Accuracy (MDA)
XGBoost	50	Gain, Cover, Frequency

**Table 2:** Performance of machine-learning models based on the six-gene signature across the training cohort and four external validation cohorts.

Algorithm Category	Algorithm	GSE213621 (NAFLD-Training, 10-Fold CV)	GSE49541 (NAFLD)	GSE84044 (HBV)	GSE130970 (NAFLD)	GSE276114 (Multi-Etiology)	Mean AUC (±SD)	Rank
Regularized Linear Models	Ridge Regression	0.890	0.942	0.838	0.915	0.914	0.900 ± 0.039	1
Regularized Linear Models	Elastic Net	0.890	0.941	0.837	0.914	0.909	0.898 ± 0.039	2
Regularized Linear Models	LASSO	0.890	0.941	0.837	0.913	0.908	0.898 ± 0.039	3
Linear Classifiers	Linear Discriminant Analysis	0.889	0.942	0.833	0.914	0.909	0.897 ± 0.041	4
Linear Classifiers	Logistic Regression	0.889	0.937	0.837	0.918	0.906	0.897 ± 0.038	5
Linear Classifiers	SVM (Linear Kernel)	0.889	0.936	0.836	0.912	0.901	0.895 ± 0.037	6
Stepwise Models	Stepwise Logistic Regression	0.890	0.933	0.834	0.912	0.899	0.894 ± 0.037	7
Ensemble Learning	Random Forest	0.852	0.813	0.800	0.794	0.823	0.817 ± 0.023	8
Ensemble Learning	XGBoost	0.877	0.682	0.812	0.500	0.831	0.740 ± 0.153	9
Ensemble Learning	Gradient Boosting Machine	0.871	0.656	0.785	0.500	0.838	0.730 ± 0.152	10

Notes: Values represent AUCs for the training cohort (GSE213621, stratified 10-fold cross-validation) and four independent validation cohorts (GSE49541, GSE84044, GSE130970, and GSE276114). “Mean AUC (±SD)” denotes the mean and standard deviation calculated across these five AUC values (training CV + four external cohorts) for each algorithm.

**Table 3:** Expression distribution data of the six-gene signature across cell types in GSE136103 (percentage of total expression).

Gene	Group	T/NK Cells	Myeloid Cells	Fibroblasts	Endothelial Cells	Hepatocytes	Cholangiocytes	B/Plasma Cells	Epithelial Cells
COL25A1	Health	0.75	0.63	51.50	3.88	24.17	20.03	0.00	0.00
	Liver fibrosis	0.00	0.00	98.64	1.36	0.00	0.00	0.00	0.00
PAPPA	Health	2.07	0.00	80.68	0.54	0.00	13.43	3.11	0.00
	Liver fibrosis	0.15	0.05	15.27	0.06	0.40	0.85	0.00	83.21
NALCN	Health	2.15	2.19	33.64	20.11	38.14	1.96	0.00	0.00
	Liver fibrosis	0.33	1.46	81.78	4.93	0.00	4.00	0.00	6.90
ITGBL1	Health	0.81	0.47	97.26	1.46	0.00	0.00	0.27	0.00
	Liver fibrosis	0.08	0.34	93.53	0.35	0.70	0.21	0.10	4.84
CLEC4M	Health	0.44	1.13	0.86	93.13	2.19	1.15	0.70	0.00
	Liver fibrosis	2.32	3.36	2.45	59.79	0.00	1.67	1.14	26.96
PEG3	Health	0.00	0.66	11.21	2.35	3.57	2.88	0.00	79.32
	Liver fibrosis	0.00	0.22	38.75	4.36	0.00	5.08	0.00	51.43

**Table 4:** Molecular docking summary of Withaferin A against proteins encoded by the six-gene signature.

Target Protein	Binding Free Energy (kcal/mol)	Key Interaction Sites	Interaction Mode and Priority
NALCN	−9.9	ILE-224 (2.3 Å H-bond), SER-1066, ASN-1070	Highest affinity. Dominated by a single strong H-bond, suggesting the most probable primary target.
CLEC4M	−8.9	GLN-297, ARG-300, LYS-390, GLN-225 (5 H-bond network)	High stability. Multi-point H-bond network indicates robust binding; a high-potential target.
PEG3	−8.9	TRP-37 (hydrophobic/π-π stacking), other H-bonds (3.0–3.2 Å)	Hydrophobic-driven. High binding energy mainly from aromatic ring interactions; a key binding mode.
PAPPA	−8.6	PRO-452 (2.4 Å H-bond), THR-240 (2.5 Å H-bond)	High efficiency. “Few but strong” H-bond pattern suggests specific binding potential.
ITGBL1	−7.1	ASP-353 (2.3 Å H-bond)	Moderate affinity. Significantly weaker binding; likely a secondary or auxiliary target.
COL25A1	−6.4	SER-398, GLU-404 (weak H-bonds)	Weak affinity. Loose binding mode suggests non-specific interaction.

Note: More negative predicted binding free energy indicates stronger theoretical affinity in docking simulations. H-bond, hydrogen bond.

**Table 5:** Dataset collection.

Dataset	Platforms	Technology	Samples	Fibrosis Stage *	Etiology	Healthy Controls	Species
GSE213621	GPL16791	RNA-seq	368	health + mild (*n* = 273), advanced (*n* = 95)	NAFLD	69	Human
GSE49541	GPL570	Microarray	72	mild (*n* = 40), advanced (*n* = 32)	NAFLD	0	Human
GSE84044	GPL570	Microarray	124	mild (*n* = 96), advanced (*n* = 28)	HBV	0	Human
GSE130970	GPL16791	RNA-seq	78	health + mild (*n* = 62), advanced (*n* = 16)	NAFLD	6	Human
GSE276114	GPL24676	RNA-seq	177	mild (*n* = 39), advanced (*n* = 138)	Multi-etiology ^#^	0	Human
GSE139602	GPL13667	Microarray	39	Different liver disease stages	Multi-etiology	6	Human
GSE136103	GPL20301	scRNA-seq	10	Health (*n* = 5); Fibrosis (*n* = 5)	Multi-etiology	5	Human
GSE172492	GPL24247	scRNA-seq	5	Health (*n* = 1); Fibrosis (*n* = 4)	CCl4-induced	1	Mus musculus

* Note: In cohorts annotated using the Scheuer histological staging system (S staging) [66,67], S0–S2 was mapped to mild fibrosis (F0–F2) and S3–S4 to advanced fibrosis (F3–F4). ^#^ Note: The cohort comprised patients with chronic viral hepatitis (CVH), alcohol-related liver disease (ARLD), and MASLD.

## Data Availability

All datasets analyzed in this study are publicly available from the GEO database. No restrictions apply to the availability of data and materials used in this study. The original contributions presented in this study are included in the article. Further inquiries can be directed to the corresponding authors.

## Supplementary Materials

The following supporting information can be downloaded at: https://www.mdpi.com/article/10.3390/ph19030495/s1, Figure S1: Violin plots of quality-control (QC) metrics in GSE136103; Figure S2: Elbow plot for principal component (PC) selection in GSE136103; Figure S3:UMAP embedding of cells in GSE136103; Figure S4. Top marker genes for each cluster in GSE136103;Figure S5. UMAP embedding of cells in GSE172492; Figure S6. Top marker genes for each cluster in GSE172492; Figure S7. Expression distribution of the six-gene signature in GSE136103; Figure S8. CLEC4M expression distribution across cell types in GSE136103; Figure S9. COL25A1 expression distribution across cell types in GSE136103; Figure S10. ITGBL1 expression distribution across cell types in GSE136103; Figure S11. NALCN expression distribution across cell types in GSE136103; Figure S12. PAPPA expression distribution across cell types in GSE136103; Figure S13. PEG3 expression distribution across cell types in GSE136103. Table S1. The top 20 candidate compounds ranked by NCS. Supplementary Table. Processed RNA-seq expression matrix (TPM) after quality control and normalization in mouse liver tissues.

## Abbreviations

ALT, alanine aminotransferase; AST, aspartate aminotransferase; AUC, area under the receiver operating characteristic curve; BP, biological process; CCl4, carbon tetrachlo-ride; CC, cellular component; CMap, Connectivity Map; CVF, collagen volume fraction; DEGs, differentially expressed genes; ECM, extracellular matrix; FDR, false discovery rate; GBD, Global Burden of Disease; GEO, Gene Expression Omnibus; GO, Gene On-tology; H&E, hematoxylin and eosin; HBV, hepatitis B virus; HCC, hepatocellular car-cinoma; HSCs, hepatic stellate cells; KEGG, Kyoto Encyclopedia of Genes and Ge-nomes; LF, liver fibrosis; LX-2, Lieming Xu-2; MASLD, metabolic dysfunction-associated steatotic liver disease; MF, molecular function; MOA, mechanism of action; NAFLD, non-alcoholic fatty liver disease; NCS, normalized connectivity score; PCA, principal component analysis; RNA-seq, RNA sequencing; scRNA-seq, single-cell RNA sequencing; SEM, standard error of the mean; SNN, shared nearest neighbor; SVM, support vector machine; TPM, transcripts per million; UMAP, Uniform Manifold Ap-proximation and Projection; WFA, Withaferin A; XGBoost, extreme gradient boosting.
