# Supplementary material for: From a Multi-Omics Signature to a Therapeutic Candidate: Computational Prediction and Experimental Validation in Liver Fibrosis
Source: Pharmaceuticals (Basel). 2026 Mar 17;19(3):495. doi: 10.3390/ph19030495 (PMC13029774; doi:10.3390/ph19030495)
Supplement: Supplementary file 1 [file pharmaceuticals-19-00495-s001.zip › Supplementary figures.pdf]

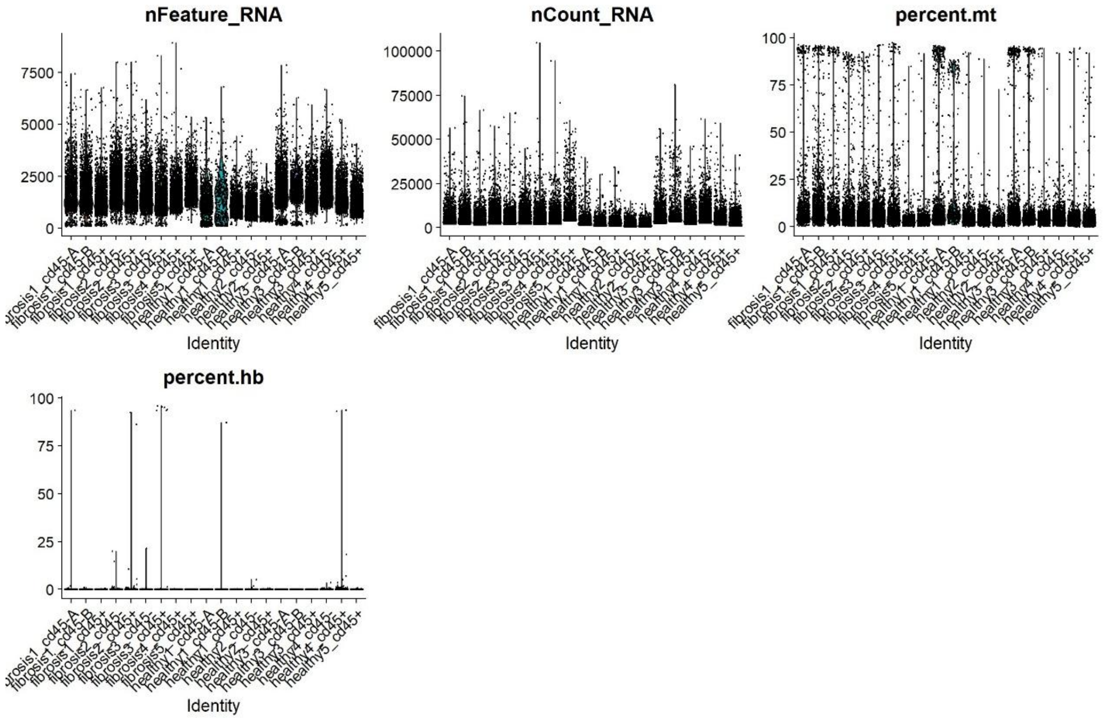

Supplementary Figure S1. Violin plots of quality-control (QC) metrics in GSE136103.

Violin plots show distributions of nFeature_RNA, nCount_RNA, mitochondrial read percentage (percent.mt), and hemoglobin read percentage (percent.hb) across cells prior to filtering. Dashed lines indicate the thresholds used for cell filtering (300 ⩽ nFeature_RNA ⩽ 7000, percent.mt < 20%, percent.hb < 3%, nCount_RNA < 100,000)

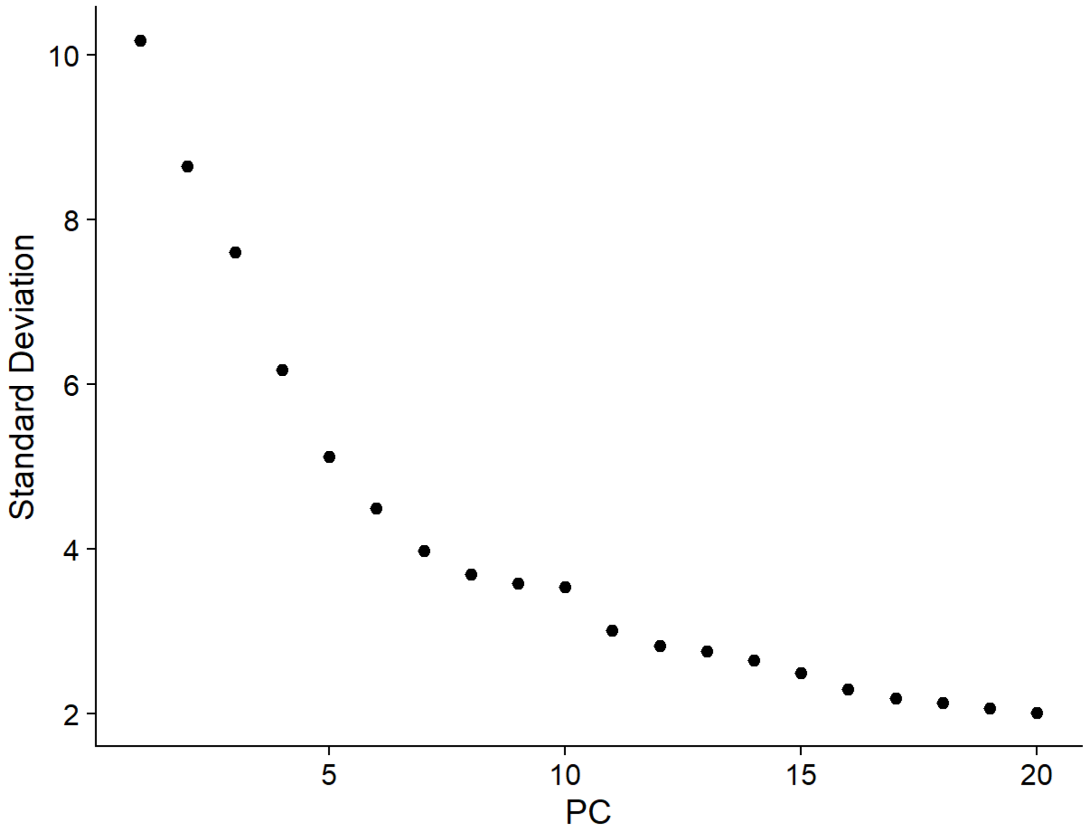

Supplementary Figure S2. Elbow plot for principal component (PC) selection in GSE136103. ElbowPlot displays the standard deviation explained by each PC after SCTransform normalization and PCA. PCs selected for downstream neighbor graph construction, clustering, and UMAP are indicated.

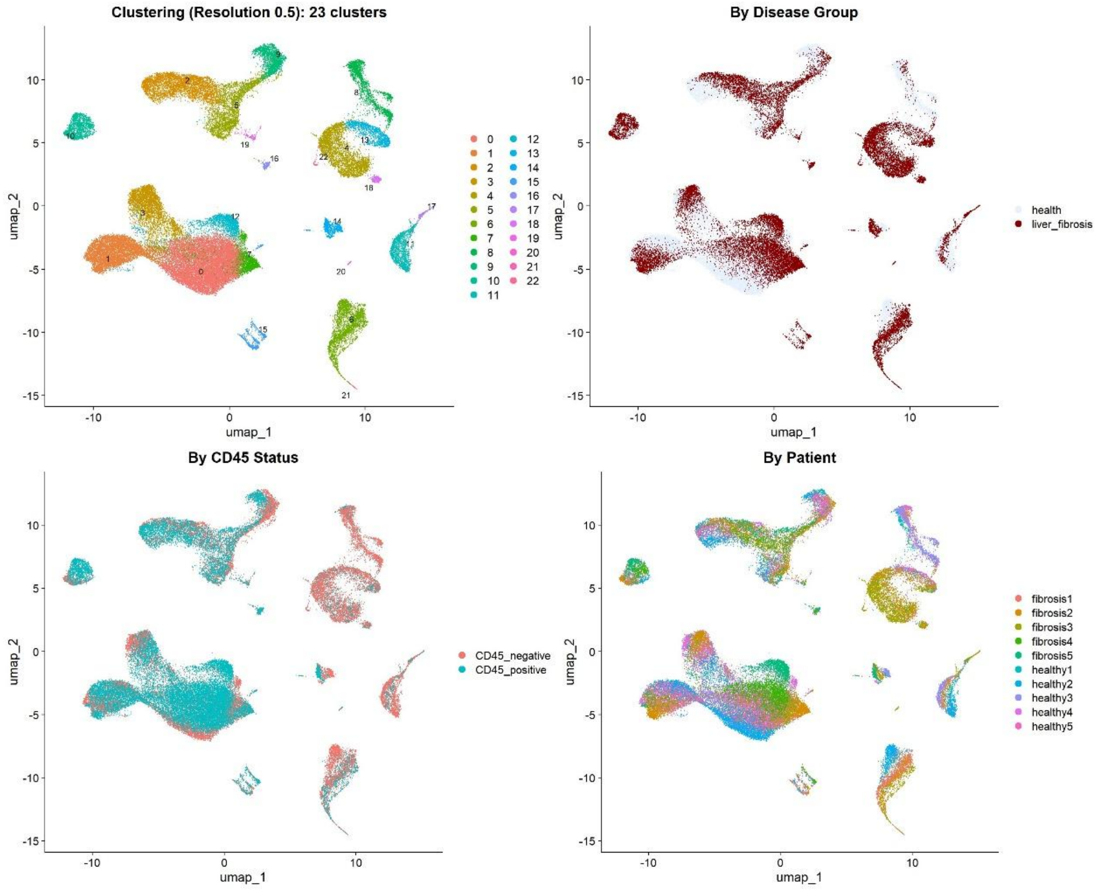

Supplementary Figure S3. UMAP embedding of cells in GSE136103. UMAP visualization of high-quality cells after QC and integration, colored by cluster identity (Seurat resolution = 0.5; neighbors and UMAP computed using PCs 1–20).

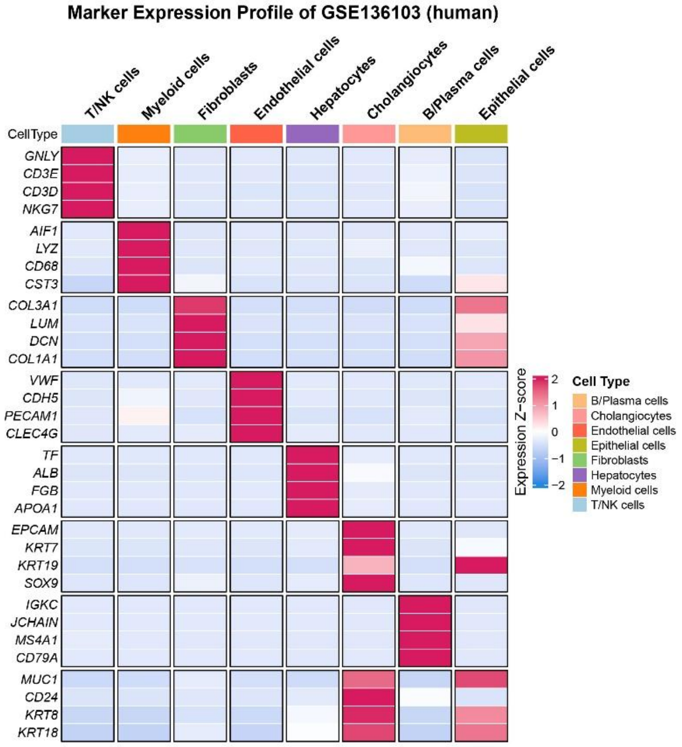

Supplementary Figure S4. Top marker genes for each cluster in GSE136103. Heatmap showing the top marker genes (top 4 per cluster) identified by differential expression testing, used for major cell-type annotation.

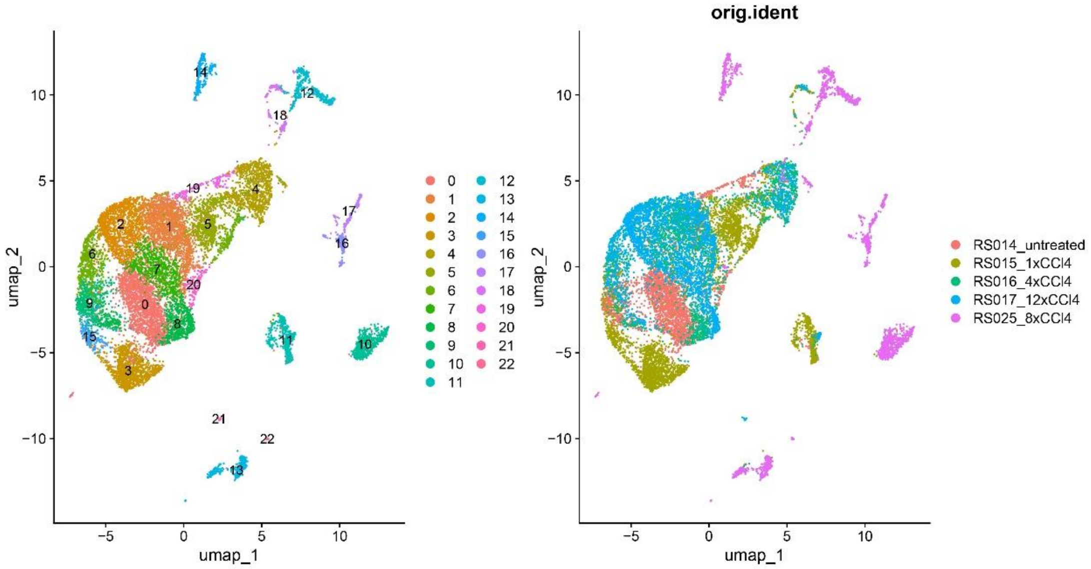

Supplementary Figure S5. UMAP embedding of cells in GSE172492. UMAP visualization of single cells in the mouse dataset GSE172492, colored by cluster identity and group.

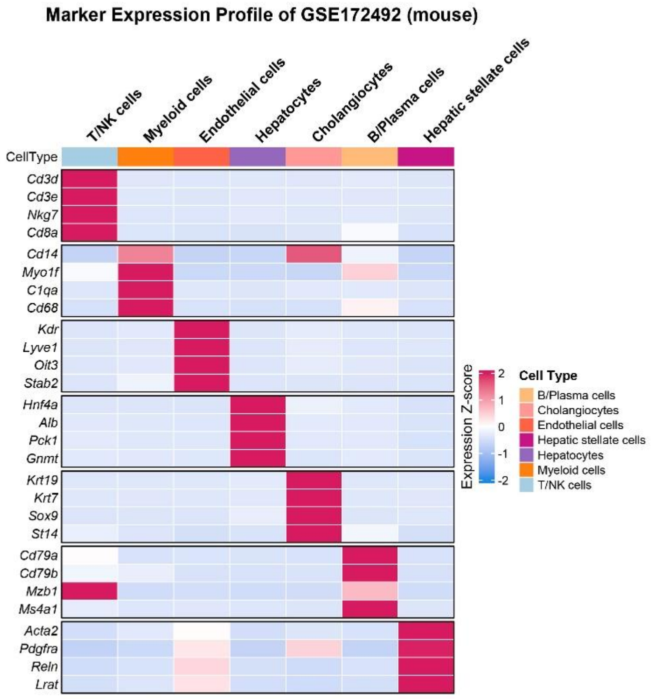

Supplementary Figure S6. Top marker genes for each cluster in GSE172492. Heatmap showing the top marker genes (top 4 per cluster) identified by differential expression testing, used for major cell-type annotation.

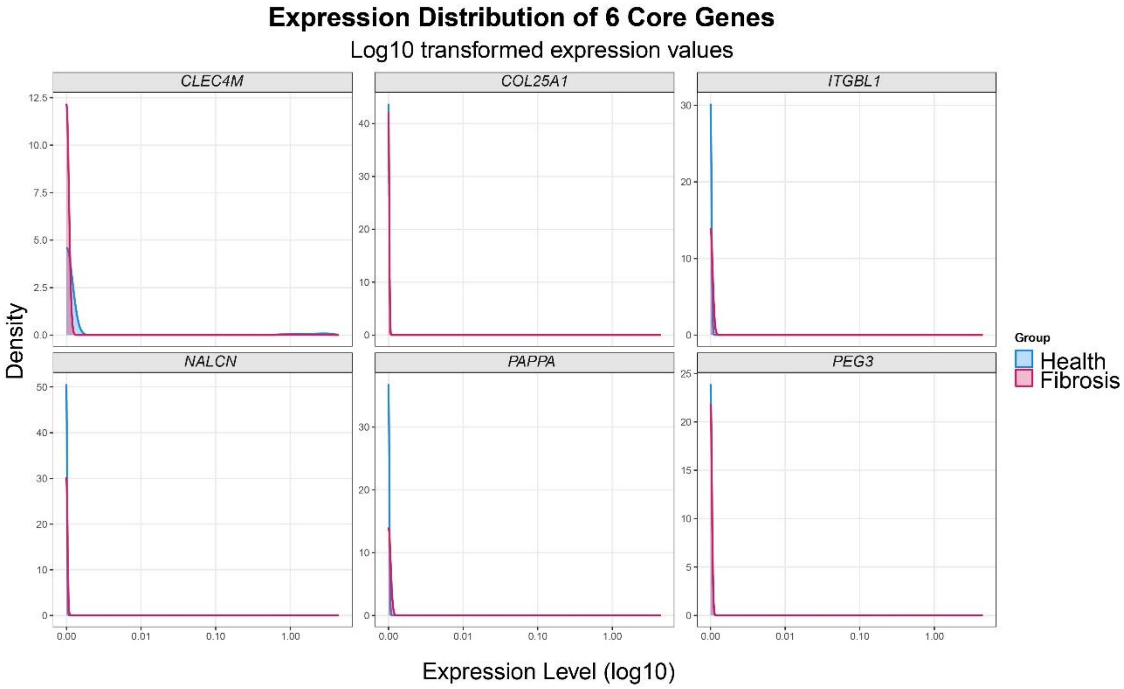

Supplementary Figure S7. Expression distribution of the six-gene signature in GSE136103. Density plot showing the log10-transformed expression values of CLEC4M, COL25A1, ITGBL1, NALCN, PAPPA, and PEG3 across all cells from healthy controls (n=5) and liver fibrosis patients (n=5).

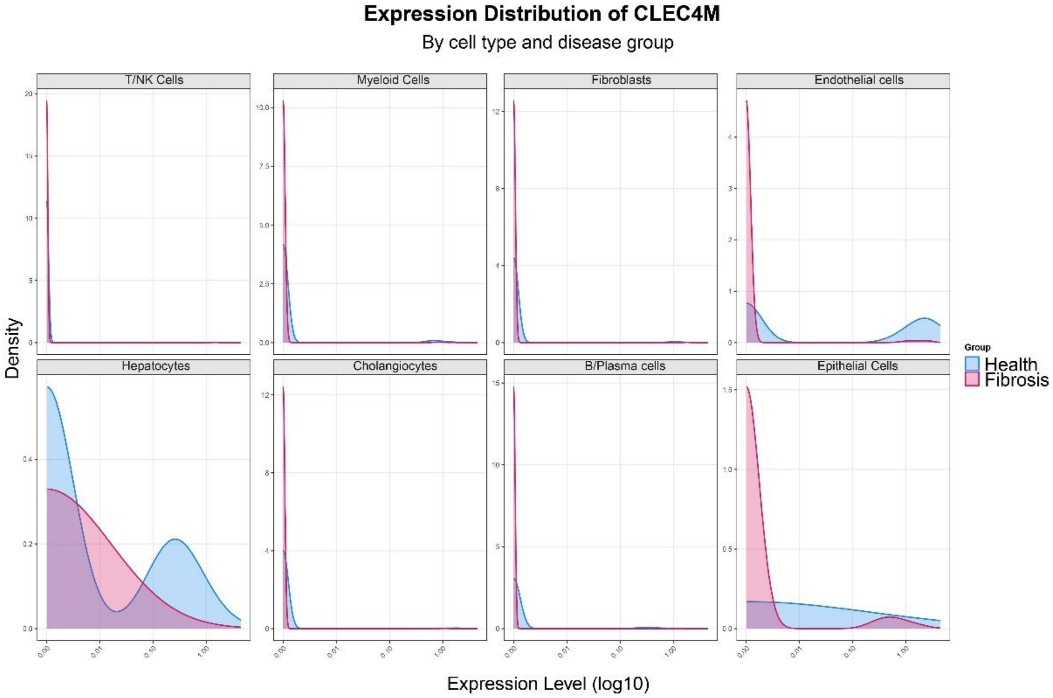

Supplementary Figure S8. CLEC4M expression distribution across cell types in GSE136103. Density plots showing log10-transformed CLEC4M expression levels in eight major liver cell populations (T/NK cells, myeloid cells, fibroblasts, endothelial cells, hepatocytes, cholangiocytes, B/plasma cells, and epithelial cells) from healthy controls and fibrotic patients (GSE136103).

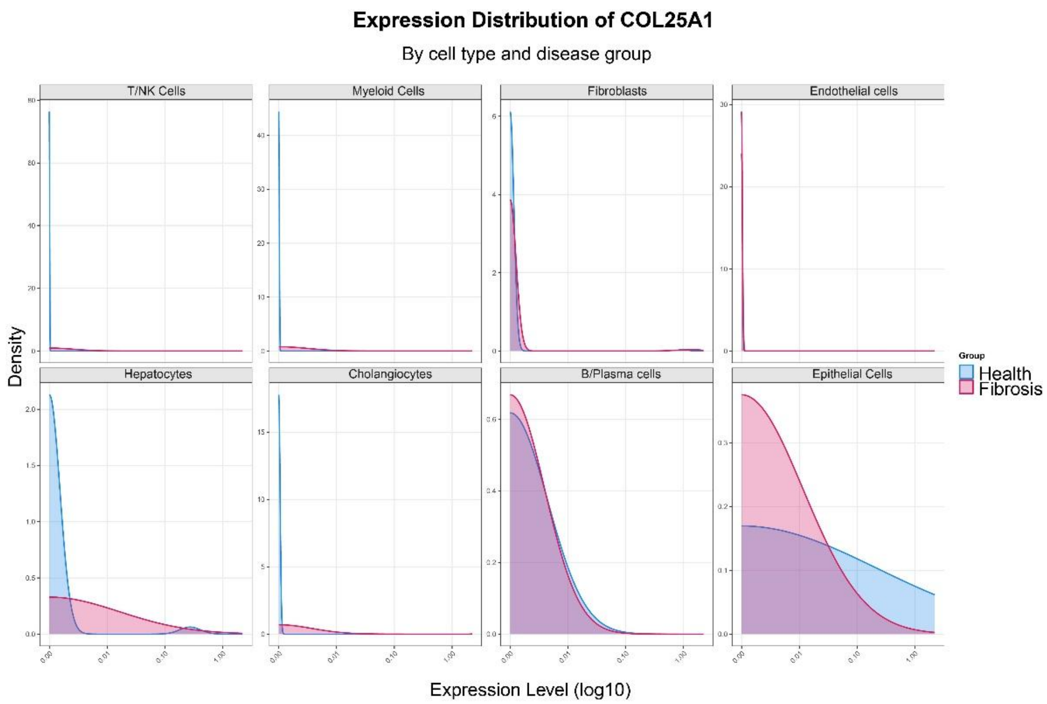

Supplementary Figure S9. COL25A1 expression distribution across cell types in GSE136103. Density plots showing log10-transformed COL25A1 expression levels in eight major liver cell populations (T/NK cells, myeloid cells, fibroblasts, endothelial cells, hepatocytes, cholangiocytes, B/plasma cells, and epithelial cells) from healthy controls and fibrotic patients (GSE136103).

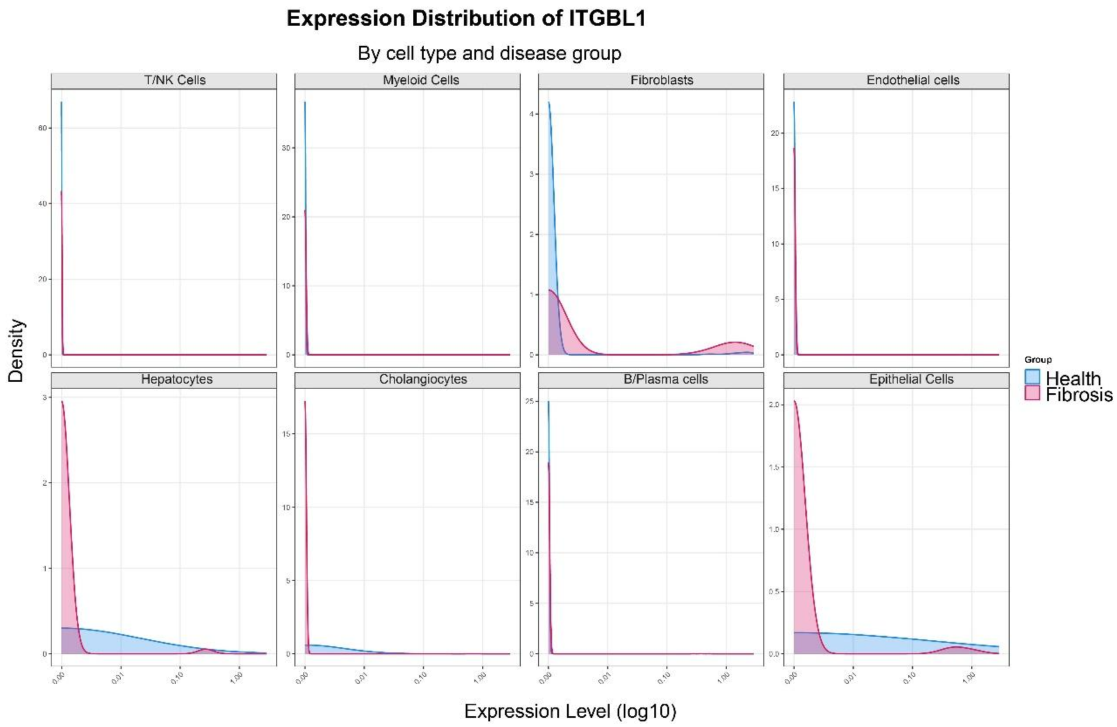

Supplementary Figure S10. ITGBL1 expression distribution across cell types in GSE136103. Density plots showing log10-transformed ITGBL1 expression levels in eight major liver cell populations (T/NK cells, myeloid cells, fibroblasts, endothelial cells, hepatocytes, cholangiocytes, B/plasma cells, and epithelial cells) from healthy controls and fibrotic patients (GSE136103).

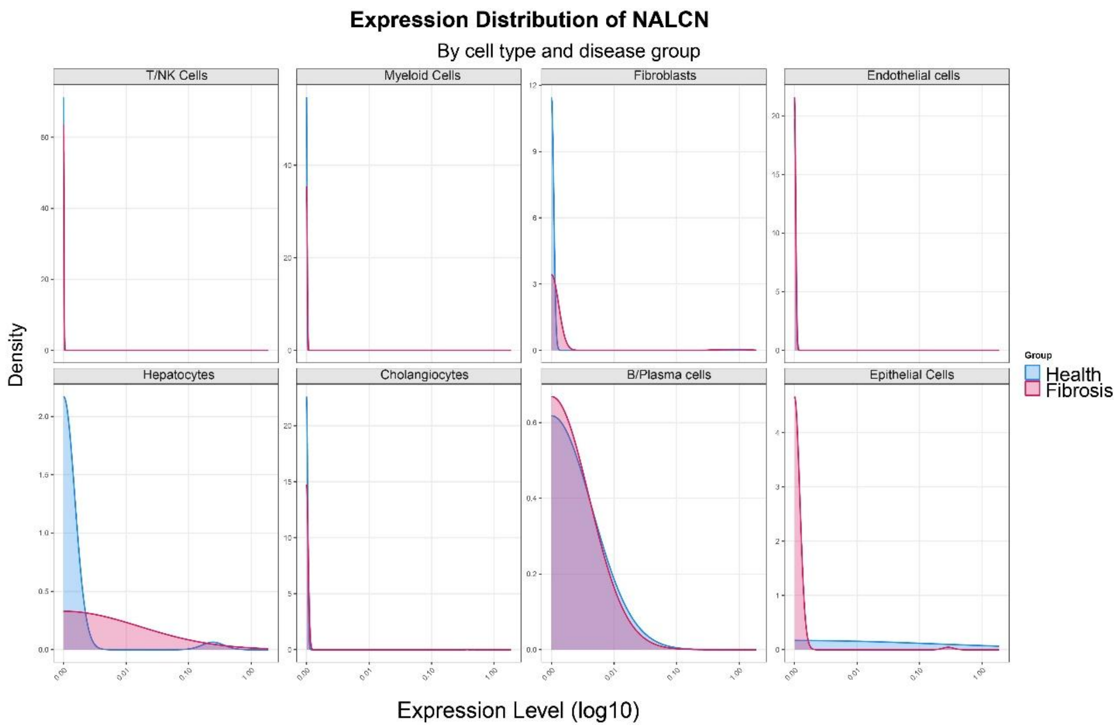

Supplementary Figure S11. NALCN expression distribution across cell types in GSE136103.
Density plots showing log10-transformed NALCN expression levels in eight major liver cell populations (T/NK cells, myeloid cells, fibroblasts, endothelial cells, hepatocytes, cholangiocytes, B/plasma cells, and epithelial cells) from healthy controls and fibrotic patients (GSE136103).

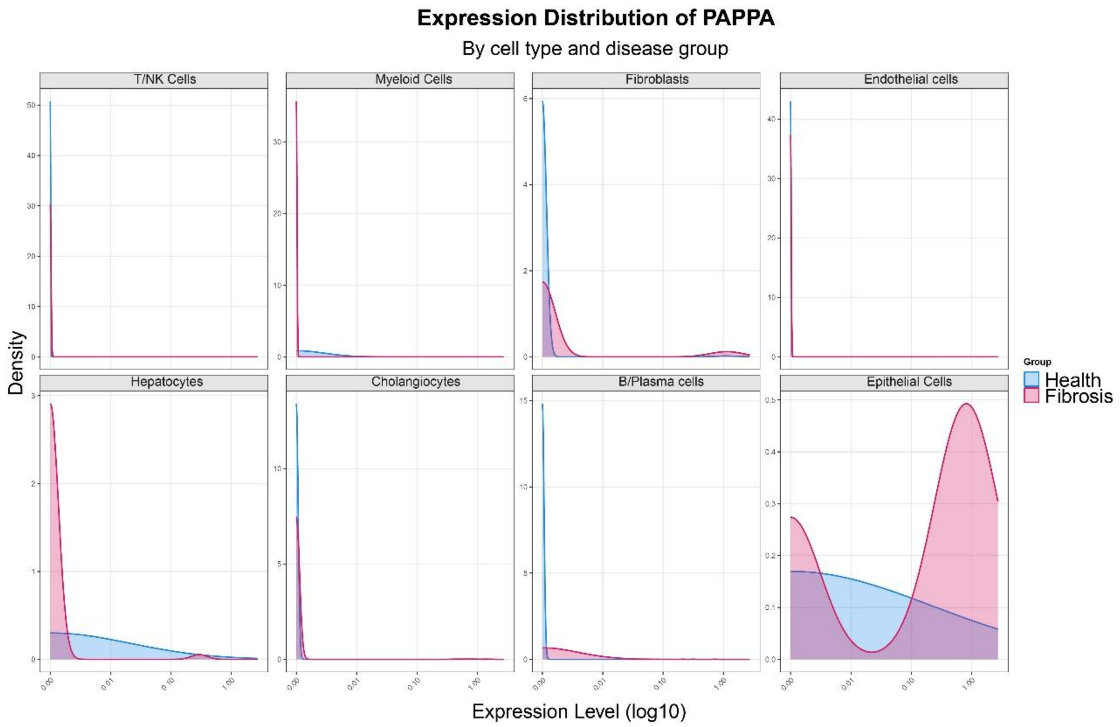

Supplementary Figure S12. PAPPA expression distribution across cell types in GSE136103.
Density plots showing log10-transformed PAPPA expression levels in eight major liver cell populations (T/NK cells, myeloid cells, fibroblasts, endothelial cells, hepatocytes, cholangiocytes, B/plasma cells, and epithelial cells) from healthy controls and fibrotic patients (GSE136103).

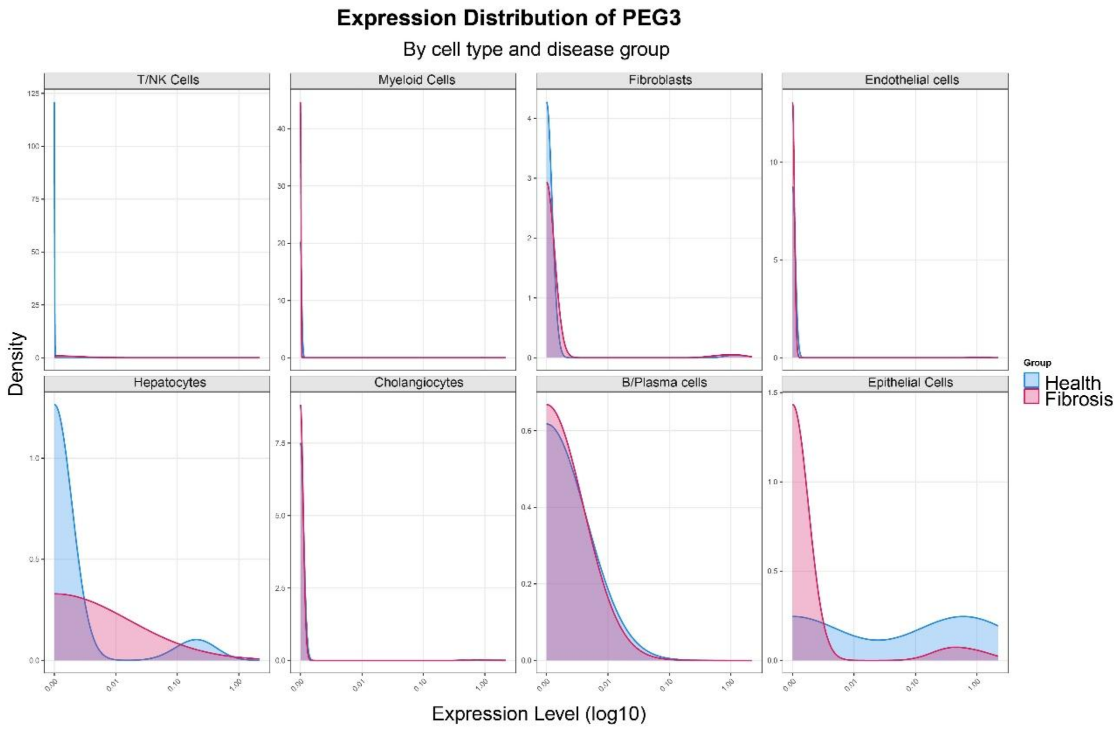

Supplementary Figure S13. PEG3 expression distribution across cell types in GSE136103.

Density plots showing log10-transformed PEG3 expression levels in eight major liver cell populations (T/NK cells, myeloid cells, fibroblasts, endothelial cells, hepatocytes, cholangiocytes, B/plasma cells, and epithelial cells) from healthy controls and fibrotic patients (GSE136103).
