# Supplementary material for: From a Multi-Omics Signature to a Therapeutic Candidate: Computational Prediction and Experimental Validation in Liver Fibrosis
Source: Pharmaceuticals (Basel). 2026 Mar 17;19(3):495. doi: 10.3390/ph19030495 (PMC13029774; doi:10.3390/ph19030495)
Supplement: Supplementary file 1 [file pharmaceuticals-19-00495-s001.zip › Supplementary Table S1. The top 20 candidate compounds ranked by NCS.pdf]

Supplementary Table S1. The top 20 candidate compounds ranked by NCS

Rank	Compound	CMap ID	NCS	False discovery rate (FDR) q-value	Cell_Line	MOA	Target
1	SAR-245409	BRD-K75308783	-2.007069111	0	NEU	PI3K inhibitor	MTOR|PIK3CG
2	tolvaptan	BRD-A82035391	-1.997337937	0	MDAMB231	Vasopressin receptor antagonist	AVPR2|AVPR1A
3	neratinib	BRD-K85606544	-1.987828493	0	HUVEC	EGFR inhibitor	EGFR|ERBB2|ERBB4|KDR
4	alvespimycin	BRD-K23499943	-1.977978587	0	HA1E	HSP inhibitor	HSP90AA1
5	levonorgestrel	BRD-K35189033	-1.916345716	0	PC3	Estrogen receptor agonist|Glucocorticoid receptor antagonist|Progesterone receptor agonist|Progesterone receptor antagonist	PGR|CYP2E1|AR|ESR1|SRD5A1

6	nicotine	BRD-K05395900	-1.913268685	0	A549	Acetylcholine receptor agonist	CHRNA10	CHRNA4	CHRNA9	CHRNB2	AOX1	CYP2B6	CHAT	CHRNA2	CHRNA3	CHRNA5	CHRNA6	CHRNA7	CHRNB3	CHRNB4	CYP19A1	TBXAS1	TRPA1
7	zalcitabine	BRD-K85925969	-1.902106881	0	HUVEC	Nucleoside reverse transcriptase inhibitor	-*
8	drospirenone	BRD-K04394237	-1.896153331	0	T47D	Mineralocorticoid receptor antagonist	NR3C2	PGR	AR
9	NVP-BGJ398	BRD-K42728290	-1.889722347	0	MCF7	FGFR inhibitor	FGFR1	FGFR2	FGFR3	FGFR4	KDR
10	fasudil	BRD-K76617868	-1.873380542	0	JURKAT	Rho associated kinase inhibitor	ROCK1	ROCK2	PKIA	PRKACA
11	withaferin-a	BRD-K88378636	-1.850906014	0	SKBR3	IKK inhibitor	IKBKB	NAMPT
12	losartan	BRD-K76205745	-1.84213841	0	H1299	Angiotensin receptor antagonist	AGTR1

13	BRD-K68336408	BRD-K68336408	-1.841804385	0	MCF7	EGFR inhibitor	EGFR丨MAPK14
14	lenvatinib	BRD-K39974922	-1.838473082	0	JURKAT	FGFR inhibitor丨KIT inhibitor丨PDGFR inhibitor丨VEGFR inhibitor	KDR丨FLT4丨FGFR1丨FLT1丨KIT丨FGFR2丨FGFR3丨FGFR4丨PDGFB丨PDGFRB丨RET
15	isbufylline	BRD-K06762493	-1.836411476	0	MDAMB231	Phosphodiesterase inhibitor	-
16	lidamidine	BRD-K68693535	-1.834986448	0	HUVEC	Adrenergic receptor agonist	-
17	ATN-161	BRD-K00521308	-1.834208488	0	HUVEC	Integrin inhibitor	ITGA5丨ITGAV丨ITGB1丨ITGB3丨ITGB5
18	olmesartan-medoxomil	BRD-K78485176	-1.834206223	0	THP1	Angiotensin receptor antagonist	AGTR1
19	MK-2461	BRD-K56405753	-1.831629992	0	MDAMB231	FGFR inhibitor丨VEGFR inhibitor	FGFR1丨FGFR2丨FGFR3丨FLT1丨FLT3丨MET丨FLT4丨KDR丨MERTK丨MST1R丨NTRK1丨NTRK2丨PDGFRB

20	lomerizine	BRD-K62858456	-1.829750896	2.22E-16	HEC108	Calcium channel blocker	ABCB1|CACNA1B|SCN5A

*Note: “-” indicates that no target information is available for the compound in the CMap/LINCS database.
