# Supplementary figures and images for: From a Multi-Omics Signature to a Therapeutic Candidate: Computational Prediction and Experimental Validation in Liver Fibrosis
Source: Pharmaceuticals (Basel). 2026 Mar 17;19(3):495. doi: 10.3390/ph19030495 (PMC13029774; doi:10.3390/ph19030495)

FN1 shown in Figure 6C

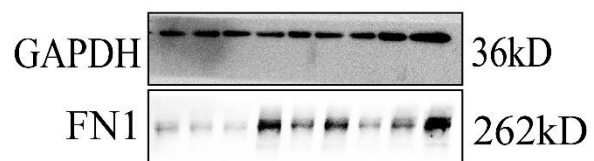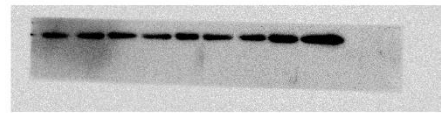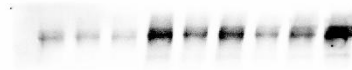

$\alpha$ -SMA shown in Figure 6C

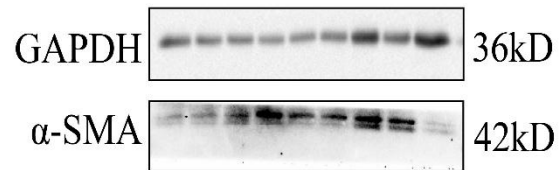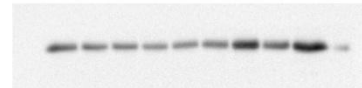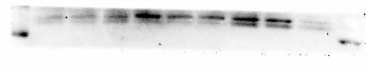

Supplement: Supplementary file 1 [file pharmaceuticals-19-00495-s001.zip › Original Images for western blotting.pdf]
